# Multicomponent SF_6_ decomposition product sensing with a gas-sensing microchip

**DOI:** 10.1038/s41378-021-00246-1

**Published:** 2021-03-01

**Authors:** Jifeng Chu, Aijun Yang, Qiongyuan Wang, Xu Yang, Dawei Wang, Xiaohua Wang, Huan Yuan, Mingzhe Rong

**Affiliations:** State Key Laboratory of Electrical Insulation and Power Equipment, Xi’an Jiaotong University, 710049 Xi’an, China

**Keywords:** Electrical and electronic engineering, Nanoscale materials

## Abstract

A difficult issue restricting the development of gas sensors is multicomponent recognition. Herein, a gas-sensing (GS) microchip loaded with three gas-sensitive materials was fabricated via a micromachining technique. Then, a portable gas detection system was built to collect the signals of the chip under various decomposition products of sulfur hexafluoride (SF_6_). Through a stacked denoising autoencoder (SDAE), a total of five high-level features could be extracted from the original signals. Combined with machine learning algorithms, the accurate classification of 47 simulants was realized, and 5-fold cross-validation proved the reliability. To investigate the generalization ability, 30 sets of examinations for testing unknown gases were performed. The results indicated that SDAE-based models exhibit better generalization performance than PCA-based models, regardless of the magnitude of noise. In addition, hypothesis testing was introduced to check the significant differences of various models, and the bagging-based back propagation neural network with SDAE exhibits superior performance at 95% confidence.

## Introduction

SF_6_ is a filling medium in electrical devices for insulation and arc extinguishing. When gas-insulated switchgear (GIS) has been working for a long time, some internal defects can still lead to partial discharge (PD). Then, the SF_6_ in GIS equipment can be decomposed to SF_5_, SF_4_, SF_3_, and so on^1^. Furthermore, these low-fluorine sulfides react with trace moisture and oxygen, thus producing multiple decomposition products (SO_2_, SO_2_F_2_, and SOF_2_)^2,3^. If arc discharge or partial overthermal (POT) faults occur, the interaction of SF_6_ with water and oxygen produces H_2_S^4,5^. Some evidence suggests that these decomposition products can reflect the running state of electrical devices^6,7^. Therefore, it is of great significance to accurately monitor these gases (H_2_S, SO_2_F_2_, SOF_2_, and SO_2_) for fault diagnosis. Many techniques have been proposed to detect SF_6_ decomposition, including infrared absorption spectrometry^8^, photoacoustic spectroscopy^9^, and gas chromatography-mass spectrometry^10^. Compared to the above three offline testing methods, gas sensors are inexpensive, small, and easily integrated; moreover, they have the potential to realize online monitoring^11–14^.

Previous research has mainly focused on improving gas-sensitive materials^15–17^. Even though incredible increases in sensitivity are constantly being achieved, the ability to discriminate multicomponent gases has lagged behind^18^. To identify gas mixtures, many approaches based on gas sensors have been implemented. Exploiting the temperature modulation of the sensor is a viable strategy for quantifying gas components^19–21^. Thus, we utilized short-period dynamic thermal modulation to acquire more information from gas sensors^22^ and established a recognition library to quantitatively detect H_2_S and SO_2_. However, this method inevitably increases the complexity and cost of the detection system. Recently, another innovative method combining gas chromatography (GC) with sensors has been promoted. Zampolli et al.^23^ developed a silicon micromachined packed column to measure benzene, toluene, and m-xylene. Broek et al.^24^ simplified a Tenax TA separation column and employed it with a sensor to quantify methanol from other interference gases. However, the effect of gas separation was obviously restricted by the filled material in the separation column, whose performance was easily influenced by the ambient temperature and flow rate. Previous research indicated that sensor arrays were a simple, fast, and effective approach for determining gas mixtures^25–27^. Zhang et al.^28^ used three independent sensors to quantitatively distinguish formaldehyde and ammonia at concentrations from 5 to 100 ppm. Recently, scholars have focused on reducing the size of the sensor array to facilitate integration on a circuit board. With micromachining technology, specific functional patterns can be customized on silicon substrates. Güntner et al.^29^ employed a silicon-based array with four sensing areas for formaldehyde detection, with a small size of 14.22 mm. Additionally, Hu et al.^30^ fabricated a four-area chip (1 cm^2^) to discriminate gases. In the field of SF_6_ decomposition gas detection, a small-volume sensor has the potential to be embedded into equipment for online monitoring, which can greatly improve production efficiency.

It is known that the number of sensory neurons in the nasal cavity is limited, yet the nose has the ability to discriminate thousands of odors. The basis for the brain to correctly discriminate odors is that each odor activates a different combination of sensory neurons^31,32^. With a function similar to that of animals’ noses, the sensor array is called an “electronic nose”. The differences in the signals of the array are used to discriminate samples. However, if only a small difference exists in the signals, the recognition may fail. Thus, it is important to extract sufficient information from the signals. In most previous research^28,33^, only the response value was extracted from the sensor signal, which actually wasted extensive powerful features. Lundström et al.^34^ used all available transient features as descriptors to reduce the dimensionality, which inspired us to extract more initial features from sensor signals.

Generally, an initial data set comprising several features requires dimensionality reduction processing to improve the sample density and distance calculation. Additionally, samples can be denoised to some extent. Most studies employ principal component analysis (PCA) for dimensionality reduction^35,36^. However, this method is a linear mapping, which may make it unsuitable for nonlinear tasks. An autoencoder (AE) is an artificial neural network that can efficiently represent the input data via unsupervised training^37^. The path from the input layer to the efficient presentation layer is called the coding process, and that from the efficient presentation layer to the output layer is called the decoding process. When the dimension of the efficient presentation layer is smaller than the input layer, dimensionality reduction is realized. Since the neural network is a nonlinear model, it is possible for the autoencoder to deal with nonlinear tasks. In addition, the autoencoder has many variants. For example, a stack autoencoder (SAE) and denoising autoencoder (DAE) can jointly constitute a stack denoising autoencoder (SDAE)^38,39^. In theory, the high-level features extracted by an SDAE have strong antinoise and generalization abilities.

In the field of gas recognition, generally, the smallest possible device and the simplest method are used to obtain the highest possible accuracy. This work is based on the use of micromachining to fabricate a gas-sensing microchip with a microthermal layer and multiple material loading regions. Compared with traditional sensors, this chip has a much smaller volume, while a stable operation is ensured. Three gas-sensitive materials (ZIF8-WO_3_, ZIF8-SnO_2_, and ZIF8-In_2_O_3_) prepared by electrospinning were coated on the sensitive regions of the GS microchip. From the signal of each sensitive area, six features were extracted, the response value, the response time, the recovery time, the time at which the maximum response is reached, and the times at which the change in the resistance is the fastest during the response and recovery stages. A total of 18 attributes from three regions constituted the original data set. Then, six machine learning algorithms were applied to cross-validate 47 simulants of SF_6_ decomposition products (H_2_S, SO_2_, SO_2_F_2_, and SOF_2_), and hypothesis testing was used to check the significant differences. Moreover, this work introduced noisy unknown data sets to compare the antinoise and generalization abilities of SDAE-based and PCA-based models.

## Results and discussion

**Fig. 1:**
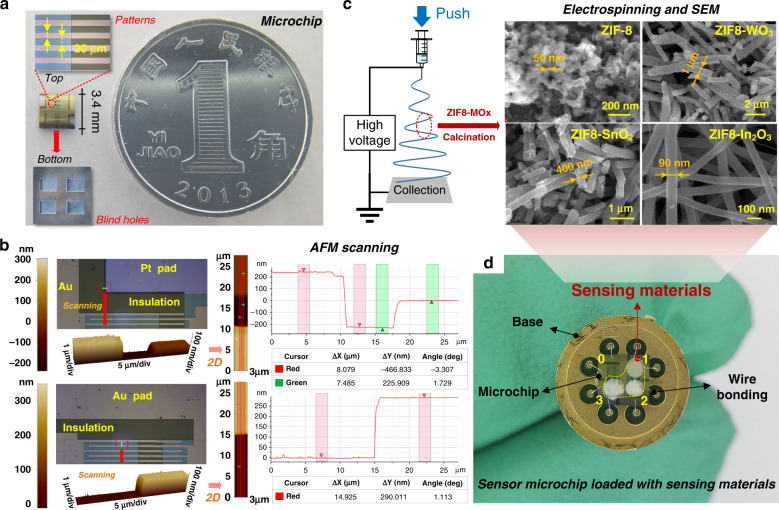
Performance of the GS microchip. **a** Optical image of the blank GS microchip. **b** AFM characteristics at various connections. **c** Schematic of the electrospinning process for ZIF8-decorated metal oxide nanowires and SEM images of ZIF8, ZIF8-WO_3_, ZIF8-In_2_O_3_, and ZIF8-SnO_2_. **d** Three sensing materials dispensed and mounted on the GS microchip bonded to the test base

The entire GS microchip was composed of a supporting substrate, microheater, insulating layer, and test electrodes. An optical image of the final blank chip is shown in Fig. 1a. The size of the chip, with four material loading regions, is just 3.4 × 3.4 mm, which is far smaller than a coin. On the front side, the illustration proves that the width and spacing of the Au electrodes are both 20 μm. On the backside, four blind square holes of 1 × 0.8 mm are assigned for thermal isolation. Figure 1b shows AFM images characterizing the sections of Pt and Au layers. The AFM scanning area is approximately 3 × 27 μm. In the upper illustration, the thickness curve is divided into three sections. It can be concluded that the thicknesses of the insulating layer and Pt heater are 466 and 225 nm, respectively, which are close to our design expectations in Supplementary Fig. S1 of the ESM. The AFM illustration below characterizes the thickness of the Au layer on the insulating layer, and the measured 290 nm is consistent with the designed 30/250 nm Cr/Au electrodes. In Fig. 1c, an electrospinning schematic and FESEM images are shown. The gas-sensing materials used in this paper are zinc-based zeolite imidazole framework (ZIF8)-anchored metal oxide nanowires. The specific synthesis is depicted in Supplementary S1.1 of the ESM. The as-prepared ZIF8 is less than 50 nm. SEM images of various composites indicate that they have obviously different diameters, ranging from 100 nm to 1 μm. It is known that a difference in morphology might result in distinct response signals that are conducive to constructing the feature matrix. Figure 1d presents the chip loading with various sensing materials. Herein, one loading region is reserved as a control group (label 0). Labels 1, 2, and 3 correspond to ZIF8-WO_3_, ZIF8-In_2_O_3_, and ZIF8-SnO_2_, respectively, as illustrated in Table 1. Finally, the chip is fixed on the base by wire bonding to facilitate performance testing.

**Table 1:** Gas-sensing materials loaded on the chip.

Region	0	1	2	3
Material	Blank	ZIF8/WO_3_	ZIF8/In_2_O_3_	ZIF8/SnO_2_

Gas sensors generally need to work at a certain temperature. For the device with a microheater, the temperature curve needs to be calibrated first. Supplementary Fig. S2a shows the temperature distribution over the GS microchip as monitored by an infrared (IR) camera at different heating voltages. To ensure the calibration accuracy, we further employ a thermocouple in addition to the IR camera. Supplementary Fig. S2b indicates the temperature-dependent voltage curve of the chip. The results prove that the temperature curves calibrated by the two means are quite close. Considering that gas-sensing materials should be operated at a certain temperature for stability, we finally apply 7 V to a Pt microheater to maintain the surface temperature at ~125 °C.

**Fig. 2:**
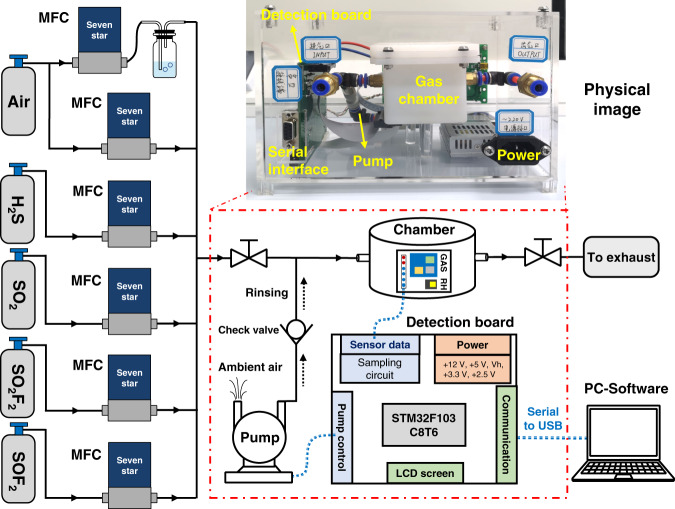
Schematic of the measurement system and portable device. The concentration of target gas is controlled by several MFCs. The portable device for field detection includes GS microchip, gas chamber, main circuit board, rinsing pump and connection module

Schematic and physical diagrams of the measurement system are shown in Fig. 2 and Supplementary Fig. S4. In the inset of Fig. 2, a portable device for field detection is illustrated. The GS microchip is the focus, but a gas chamber, main circuit board, rinsing pump, and connection module are also included. This device can transmit the signals of the GS microchip to the computer for further analysis. The detailed assembly process is performed experimentally. Apart from the portable detection device, the experimental system needs a mass-flow controller (MFC) to control the concentrations of target gases in the chamber. For the purpose of adjusting the relative humidity level, an additional MFC is connected to the air bubbler. The concentration and humidity of the target gas can be adjusted by changing the flow rates among dry air, wet air, and calibration gases. A humidity detector (TI, HDC 1080) is put into the chamber to monitor the relative humidity. Herein, six MFCs are employed for dry air, humid air, and four SF_6_ decomposition components. Without consideration of the humidity, the concentrations of the four target gases ranged from 0 to 50 ppm (Supplementary Table S1). According to various gas compositions, the 47 classification labels can be divided into four groups (test 1 to test 4). Under various humidity backgrounds (25%, 33%, 50%, and 75%), the concentrations of the four target gases range from 0 to 30 ppm (Supplementary Table S5). The exposure time and flushing time are set to 100 and 300 s, respectively. The total flow rate is set to 200 sccm.

The response value (*S*) can be calculated as follows:

1 $$S = \left( {R_{\mathrm{a}} - R_{\mathrm{g}}} \right)/R_{\mathrm{a}} \times 100\%$$

where *R*_a_ and *R*_g_ represent the sensor resistances in air and the target gas, respectively. In addition, the response time (*τ*_res) can be described as the time that elapses when there is a stepwise change in the quantity to be measured between the moment when this change starts and the moment when the indicator reaches a value conventionally fixed at 90% of the final change in indication. The definition of the recovery time (*τ*_rec) is similar to that of the response time, except that the curve changes in the opposite direction.

**Fig. 3:**
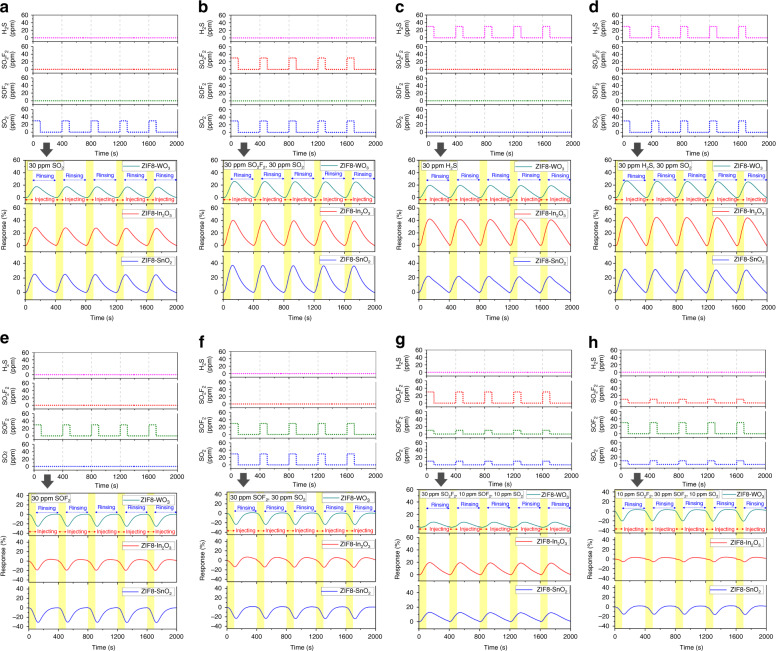
Five cycling response-recovery curves to verify the repeatability. The original signals of the GS microchip under exposure to **a** 30 ppm SO_2_, **b** 30 ppm SO_2_F_2_ and 30 ppm SO_2_, **c** 30 ppm H_2_S, **d** 30 ppm H_2_S and 30 ppm SO_2_, **e** 30 ppm SOF_2_, **f** 30 ppm SOF_2_ and 30 ppm SO_2_, **g** 30 ppm SO_2_F_2_, 10 ppm SOF_2_ and 10 ppm SO_2_, and **h** 10 ppm SO_2_F_2_, 30 ppm SOF_2_ and 10 ppm SO_2_

A stable signal of the device is the premise for its long-term operation. In Fig. 3, for exposures to eight different mixtures of SF_6_ decomposition components, five cycling response–recovery curves are presented to verify the repeatability of the output of the GS microchip. There are obvious differences in response values and response–recovery times for different measured gases, which provides the basis for further gas recognition. For instance, when exposed to 30 ppm SO_2_ (Fig. 3a), all three materials’ resistances decrease, exhibiting a positive response direction. If 30 ppm SO_2_F_2_ is injected (Fig. 3b) into 30 ppm SO_2_, the resistances of the three materials further decrease. When the target gas contains H_2_S (Fig. 3c, d), the recovery characteristics of the GS microchip are worse. This may be related to the chemical adsorption of H_2_S on the surface of the material^40^. If SOF_2_ participates in the mixtures (Fig. 3e, f), the response directions of the three modules might be negative, exhibiting an increase in the resistance. Figure 3g, h illustrates the response signals when three gas components are present. The response directions of the materials are relevant to the content of SOF_2_. The higher the content of SOF_2_, the more the response curve moves in the negative response direction, and vice versa.

After the repeatability of the experiments is confirmed, the response–recovery curves (original signals) of the MGC chip exposed to various mixtures are obtained. In this process, 15 cyclic tests are conducted under each atmosphere to construct a data set. Therefore, a final data set containing 705 samples can be achieved for 47 measured gases. According to the signal of the GS microchip, the concentrations of gas components can be calculated. By extracting the signal features, we can simplify the original signals and establish a mapping from the feature to the gas concentration.

From each original response signal, we can extract at least 6 descriptors. Taking ZIF8-In_2_O_3_ (region 2) as an example, the three common descriptors of the response value (*S*), response time (*τ*_res), and recovery time (*τ*_rec) were usually employed in most previous studies^41,42^, as shown in Supplementary Fig. S5a. Hierlemann summarized the transient parameters that can be extracted from sensor response curves^34,43^. To acquire more information from the response curve, the first derivative curves can be further analyzed to acquire another three descriptors (Supplementary Fig. S5b): the time (*τ*_smax) at which the maximum response is reached and the times at which the change in the resistance is the fastest during the response stage (*τ*_max) and recovery stage (*τ*_min). Wu et al.^44^ investigated the ozone-sensing properties of amorphous indium gallium zinc oxide and found that the maximum of the first derivative function was proportional to the concentration of ozone. Additionally, Laminack et al.^45^ proved that the first derivative is linear with the gas concentration. Thus, features can be extracted from the first derivative curve. Many studies^46,47^ extracted features from the derivative curve, such as the maximum derivative, to improve the identification accuracy of algorithms.

Each response signal from the chip can export 6 descriptors, and the formed feature matrix can be expressed as:

2 $$F = \left[ {S,\tau \_{\mathrm{res,}}\tau \_{\mathrm{rec,}}\tau \_{\mathrm{smax,}}\tau \_{\mathrm{max,}}\tau \_\min } \right]$$

**Fig. 4:**
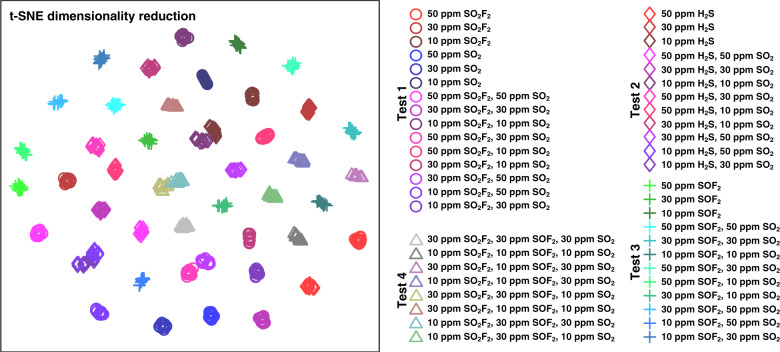
Sample visualization in two-dimensional space by t-SNE. Samples with 18 dimensions can be mapped to two-dimensional space to visualize the original dataset. Few overlapping samples mean that local features can efficiently express the original samples

Therefore, three gas-sensitive materials, providing 18 attributes in total, construct the original data set (705 samples). Considering that high dimensionality is not conducive to observing the relationship between samples, it is necessary to first reduce the dimensionality of the data set. t-distribution random neighborhood embedding (t-SNE) is a nonlinear dimensionality reduction method that can solve the congestion problem of high-dimensional data in a low-dimensional state^48,49^. For our original data set with 705 samples, the t-SNE method is utilized for clustering analysis, as shown in Fig. 4. Samples with 18 dimensions can be mapped to two-dimensional space to visualize the original data set. There are few overlapping samples, which means that local features can efficiently express the original samples. However, the problem is that t-SNE does not provide a unique optimal solution, which means it does not directly reduce the dimensionality in the test set. Moreover, t-SNE obtains the sample distance by calculating the probability distribution, and the distance is meaningless. Therefore, most researchers mainly use t-SNE for visualization, and it is difficult to use for other purposes, such as the dimensionality reduction of the test set. Therefore, we further utilize the PCA method to process the samples and observe their aggregation under different target gases.

The PCA-based visualization results for the original data set are shown in Supplementary Fig. S6, where the samples show regular clustering for different target gases. Lighter color indicates a higher concentration, and a different shape indicates different test groups (Supplementary Table S1). Samples represented by triangles are a mixture of SO_2_F_2_, SOF_2_, and SO_2_, and their clustering region expresses a high correlation with the concentration of SOF_2_. When the SOF2 concentration is increased to 30 ppm, the clustering region is closer to the group of test 3. When the SOF_2_ concentration is 10 ppm, the clustering area is closer to the group of test 1, which suggests that the effect of SO_2_F_2_ and SO_2_ is dominant.

**Fig. 5:**
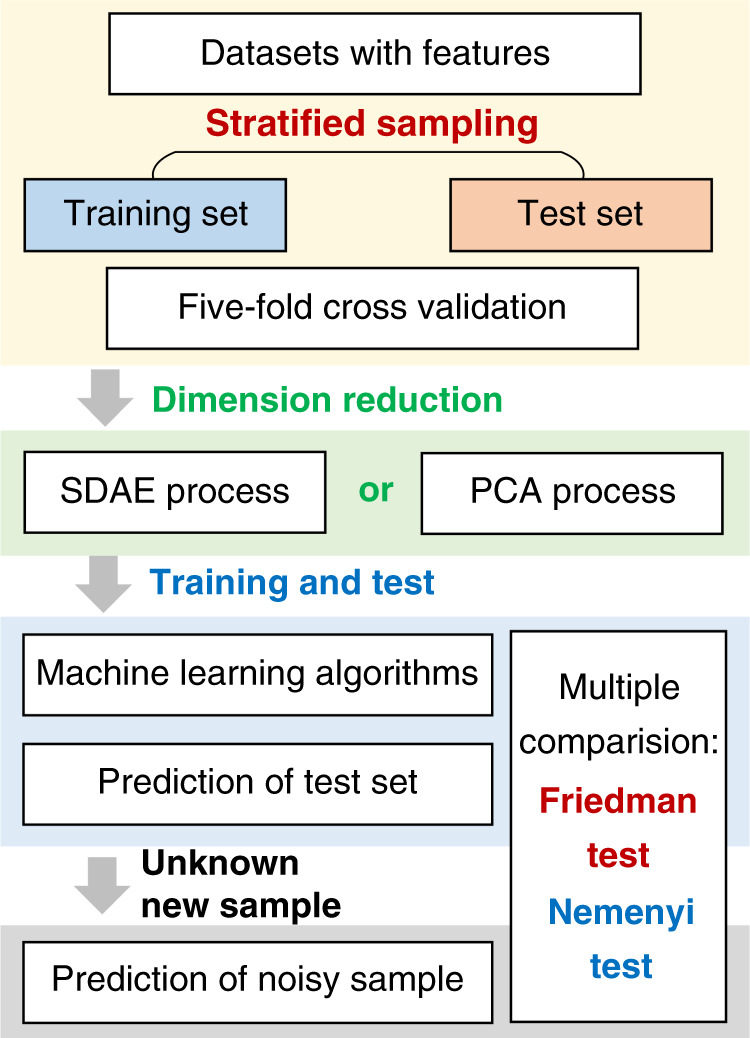
The overall flow chart of gas recognition. The original dataset is equally divided into 5 sub datasets with stratified sampling. Then, SDAE extracts the features and 6 machine learning algorithms are used to train gas recognition models. Finally, the as-trained models are applied to infer the components of the unknown gas

The overall approach to gas recognition is shown in Fig. 5. Considering the limited size of the data set, cross-validation must be introduced to assess the method’s reliability. With stratified sampling, the original data set is equally divided into 5 sub-data sets, and the split ratio between the training and test sets is 4:1. The original dimension of the data set is high at 18. To avoid dimensional disaster, it is necessary to reduce the dimensionality of the original data set. Compared to the conventional PCA linear method, SDAE dimensionality reduction can extract the features that efficiently represent the original sample. Moreover, drift is one of the inherent defects of gas sensors and may cause the signal to be noisy. Owing to its stronger antinoise potential, SDAE provides a good strategy for improving the accuracy of recognition. Combining 6 kinds of machine learning algorithms, we use various data sets from 5-fold cross-validation to train the gas recognition models. To further compare the generalization abilities of the SDAE-based and PCA-based models, the “unknown new samples” obtained in another test period are introduced. Then, the as-trained identification models are applied to infer the components of the unknown gas. To increase the persuasiveness of the test, noise with various amplitudes is artificially added to the unknown samples, followed by a comparison of the accuracy of the models. To evaluate whether the overall differences across the 12 recognition learners are statistically significant, the learner only has the difference in SDAE and PCA. Hypothesis tests, including the corrected Friedman test and the post hoc Nemenyi test, are employed.

**Fig. 6:**
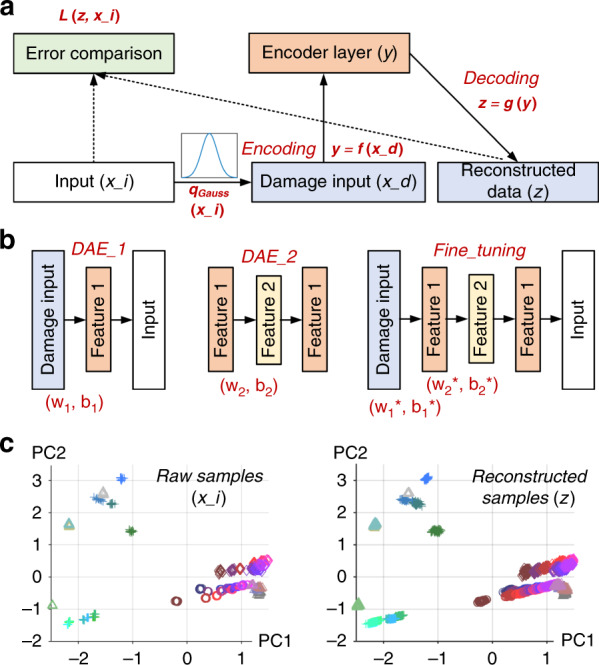
Dimensionality reduction of the original data set. The schematics of **a** DAE and **b** SDAE. **c** In PCA sample space, the raw samples and SDAE reconstructed samples

Figure 6a, b shows schematic diagrams of the denoising autoencoder (DAE) and stack denoising autoencoder (SDAE). The output layer of the DAE is the original data set (*x_i*), and its input layer is the damaged data set (*x_d*). One approach to damaging the original data set is the addition of Gaussian noise to each attribute of the sample. For the distribution of the Gaussian noise, the mean is set to zero, and the standard deviation is 10% that of the original data. During the training process, the neural network can automatically extract deep-level features that can withstand noise to some extent and achieve dimensionality reduction at the same time. Comparing the difference (*L*) between the reconstructed result of DAE and the original sample, we can acquire the reconstruction error, which determines the DAE performance.

Actually, a single-layer autoencoder only has limited dimensionality reduction capabilities, and excessive processing might lose key information of the sample. A stacked denoising autoencoder (SDAE) can solve this issue, as shown in Fig. 6b. It can be regarded as a stack of several DAEs that preserve the key features as much as possible. The layer-by-layer greedy method is used to train each DAE in turn and then pretrain the entire SDAE. Each DAE can export a set of weights and thresholds (*w*, *b*). After the pretraining is complete, the parameters of all the layers are substituted into the SDAE for final training. This step is called “fine-tuning”.

Herein, a two-layer SDAE is constructed to reduce the dimensionality of the original data and extract deep-level features. The first autoencoder (DAE_1) has 18 input neurons. After encoding and dimensionality reduction, a reduced data set containing 10 attributes is obtained. Continuously, the first reduced data set is used as the input of the second autoencoder (DAE_2), and then a second reduced data set with 5 features is obtained. Finally, the parameters of these two DAEs are substituted into the SDAE for fine-tuning. Supplementary Fig. S7 gives the SDAE training curve and the error regression curve of the first neuron in the output layer. It can be easily found that the model is continuously optimized as the iteration progresses. Observing the error regression curve, we find that the fitting level (*R*^2^) of the fine-tuning is much closer to 1 than those of DAE_1 and DAE_2, which verifies the effectiveness of fine-tuning regarding improving the SDAE performance.

After SDAE processing, the dimensionality of the original data set is reduced from 18 to 5, which realizes sample embedding from high to low dimensions and decreases the impact of dimension disaster. In theory, due to the use of SDAE, the extracted features have better antinoise abilities. Figure 6c shows the distribution of the original and reconstructed samples in PCA space. Even under the influence of slight noise, the reconstruction results are quite close to those of the raw samples. This proves that the deep-level features extracted by SDAE can efficiently represent the original sample.

After the reduced samples are acquired, gas recognition models are trained with different sub-data sets from 5-fold cross-validation, as shown in Fig. 8a. To avoid misleading conclusions derived from a single algorithm and to screen more suitable methods for gas recognition, this paper compares 6 machine learning algorithms. In addition, their principles are presented in Supplementary Figs. S8–S13. KNN, AdaBoost decision tree, and SVM are skilled in solving classification tasks, but BPNN can handle regression tasks. Combining the heuristic genetic algorithm (GA), the GA-BPNN can search for more reasonable solutions to avoid falling into local optima. The bagging-BPNN needs a bootstrapping process to sample the training set and achieves multiple sub-learners with the out-of-bag estimate. Usually, the integration of multiple sub-learners performs better than single learners, especially in generalization.

Table 2 expresses the parameter settings of different machine learning algorithms. The accuracy of KNN is mainly related to the value of neighbors (*k*). Considering that each label uses 12 samples (80%) as the training set, *k* is set to 12. The AdaBoost decision tree needs to expand binary classification to 1-versus-1 multiple classification. For a data set with 47 labels, a total of 1081 sub-classifiers (^47^C_2_) are required. The libsvm package is used to build the SVM model, and the kernel function is the most commonly used RBF. The input and output neurons of the BPNN are determined by the dimension of the input sample (5) and the component of the target gas (4), respectively. Therefore, the performance of the BPNN is mainly related to the hidden layers and neurons, which are decided by the recognition errors (Supplementary Fig. S14). A darker color indicates better performance. According to the cross-validated results of the five data sets, five recognition errors rapidly decrease as the number of neurons in the first hidden layer gradually increases. However, increasing the number of neurons in the second hidden layer does not significantly improve the performance of the network. To acquire a relatively superior model, the neuron numbers in the first and second layers are set to 12 and 8, respectively. For the GA-BPNN, the parameters of the GA, such as the iterations, size of a generation, cross probability, and mutation probability, need to be set. The bagging-BPNN uses the bootstrapping method to integrate 10 sub-learners for comprehensively calculating the gas mixtures.

**Table 2:** Parameter settings of different machine learning algorithms.

Algorithm	Parameters
KNN	Neighbors (k) equal to 12
AdaBoost decision tree	Multiclassification, 1081 sub-classifiers
SVM	Multiclassification, RBF kernel
BPNN	4 Layers ([5,12,8,4]), MSE
GA-BPNN	GA including generation (20), population size (30), crossover probability (0.4), mutation probability (0.1), 4 layers ([5,12,8,4]), MSE
Bagging-BPNN	Bootstrapping, 4 layers ([5,12,8,4]), MSE, 10 sub-classifiers

**Fig. 7:**
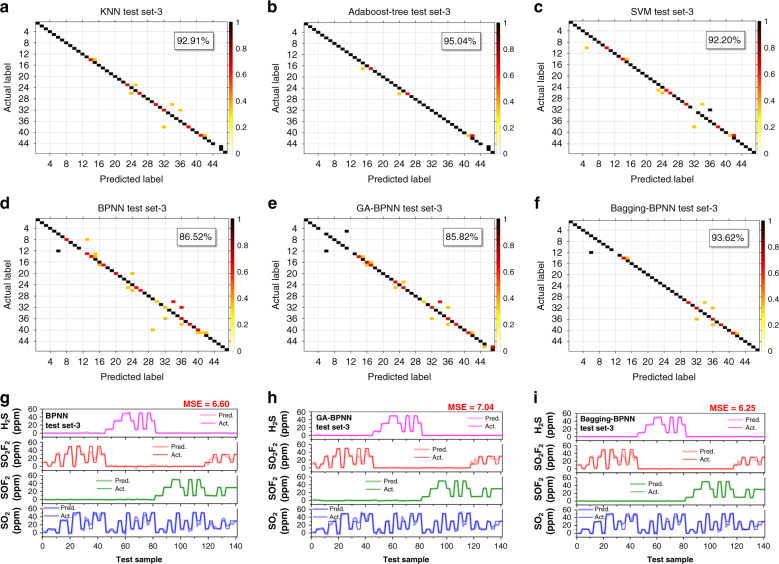
Gas recognition with machine learning algorithms. For the third cross-validation data set, the confusion matrix of **a** KNN, **b** AdaBoost decision tree, **c** SVM, **d** BPNN, **e** GA-BPNN, **f** bagging-BPNN. The regressions predicting the concentrations of gas mixtures via **g** BPNN, **h** GA-BPNN, and **i** bagging-BPNN

The test results of the different gas recognition models for the third data set of the 5-fold cross-validation are shown in Fig. 7a–f. Among the models, the Adaboost decision tree achieves the highest classification accuracy (95.04%). For BPNN, the gas concentrations are predicted by regression, and then the distance formula is used to assign the predicted classification. However, when the regression model is used to deal with the classification task, the accuracy of the BPNN is relatively low, at 86.52%. With GA processing, the accuracy slightly worse (85.82%). This can be ascribed to the fact that the heuristic method (the GA) cannot ensure a better model but might fall into a worse local optimum. The bagging method takes full advantage of integration and performs significantly better than a single BPNN. The final classification accuracy increases to 93.62%. Furthermore, we present the regression results of three models based on BPNN in Fig. 7g–i. The colors represent different gases, the light line is the predicted result, and the dark line is the actual value. A higher overlap between the predicted and actual values means that the algorithm is more accurate. From the point of view of the mean square error (MSE), the bagging-BPNN (6.25) performs better than the BPNN (6.60) and GA-BPNN (7.04).

In fact, the quantitative identification of gas components should be a regression task; thus, a neural network is more suitable. One of the reasons for using classification accuracy is to facilitate a comparison of classification and regression algorithms. The other reason is that the confusion matrix can intuitively show the recognition results. For the other four cross-validation data sets, the matrices expressing the classification accuracy are shown in Supplementary Figs. S15–S18. Other regression results of the three models based on BPNN are given in Supplementary Fig. S19.

**Fig. 8:**
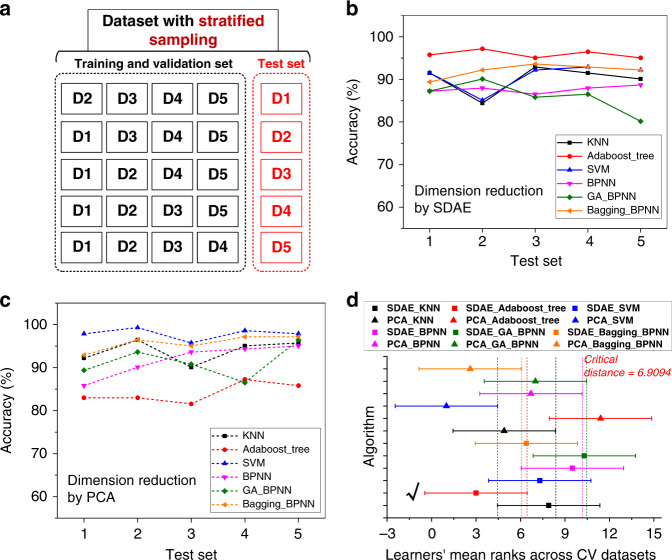
Cross-validation and hypothesis test. **a** Schematic of 5-fold cross-validation. For various cross-validation data sets, the recognition accuracies of the **b** SDAE-based models and **c** PCA-based models. **d** The result of the Nemenyi post hoc test when *α* is equal to 0.1

For various 5-fold cross-validation data sets, Fig. 8b, c shows the classification accuracies of the SDAE-based and PCA-based models, respectively. The bagging-BPNN exhibits relatively good performance regardless of which dimensionality reduction method is chosen. For the third cross-validation data set, Supplementary Table S2 gives the regression prediction results by the bagging-BPNN based on SDAE dimensionality reduction. The predicted concentrations are almost consistent with the actual values. For the SDAE-based models, the AdaBoost decision tree achieves the highest classification accuracy. For the PCA-based models, SVM exhibits the best performance.

The cross-validation results show that the classification accuracy of PCA-based models is slightly higher than that of SDAE-based models. This can be attributed to the discrepancies in the 15 original signals being too small upon exposure to the same detected gas. This also proves the stability of the sensitive materials used. When using SDAE, the introduced antinoise algorithm increases the dispersion in the data set, leading to a decrease in accuracy. However, the PCA-based models may yield better performance on the cross-validation data set, but their antinoise and generalization abilities cannot be guaranteed.

For the cross-validation data sets, the recognition results based on two-dimensionality reduction methods do not show much difference. Then, a statistical hypothesis test can be used for the comparison of algorithm performance. The Friedman test and Nemenyi post hoc test can simultaneously compare multiple algorithms based on performance ranking, and the flow chart is shown in Supplementary Fig. S20.

Based on ranking rather than actual specific performance, the Friedman test is a useful method that is less susceptible to outliers. First, it is necessary to sort the algorithms according to their performance. The best algorithm obtains the rank of 1, the second-best obtains the rank of 2, and so on. In the case of ties, the average rank is assigned. Finally, the Friedman test calculates the average rank (*r*_*i*_) of each model on all data sets, which is presented as follows^50^:

3 $$r_i = \frac{1}{N}\mathop {\sum}\limits_{j = 1}^N {R_j^i}$$

where $R_j^i$ stands for the rank of model *i* on data set *j*. *N* is the number of data sets.

The Friedman test assumes that “all approaches exhibit the same performance”. We calculate the variables following the chi-square distribution (Eq. (4)) and *F* distribution (Eq. (5)) and then compare the variables with critical values to judge whether the Friedman hypothesis is true or not.

The variable following the chi-square distribution is calculated as follows:

4 $$\Gamma \chi ^2 = \frac{{12\,N}}{{k\left( {k + 1} \right)}}\left( {\mathop {\sum}\limits_{i = 1}^k {r_i^2} - \frac{{k\left( {k + 1} \right)^2}}{4}} \right)$$

where *k* is the number of models. $\Gamma \chi ^2$ follows the chi-square distribution with (*k* − 1) degrees of freedom. Furthermore, Iman and Davenport^51^ reported that the initial Friedman statistic ($\Gamma \chi ^2$) was undesirably conservative, and they derived a corrected *F* distribution with (*k* − 1) and (*k* − 1)·(*N* − 1) degrees of freedom. The corrected statistic ($\Gamma _F$) can be defined as^52^:

5 $$\Gamma _F = \frac{{\left( {N - 1} \right)\Gamma \chi ^2}}{{N\left( {k - 1} \right) - \Gamma \chi ^2}}$$

For the five cross-validation data sets, the ranks of the 12 models are presented in Table 3. Then, the corrected Friedman statistic is employed to verify the hypothesis of whether all models exhibit the same performance. According to Eq. (4) and Eq. (5), $\Gamma _F$ can be calculated to be 13.665, which is larger than the critical value of 2.014 at the significance level (*α*) of 0.05. Thus, there is a 95% confidence that the Friedman hypothesis is not true, and the Friedman hypothesis can be overturned. Furthermore, with the Nemenyi post hoc test, we can distinguish the obvious differences among the algorithms. The Nemenyi post hoc test assumes that “the algorithms involved in the comparison are same”. If the difference between the average ranks of two algorithms exceeds the critical distance (CD), the Nemenyi hypothesis should be rejected.

**Table 3:** The ranks of all the models for the five cross-validation data sets.

Data set	SDAE-KNN	SDAE-Ada	SDAE-SVM	SDAE-BPNN	SDAE-GA	SDAE-Bag	PCA- KNN	PCA- Ada	PCA- SVM	PCA- BPNN	PCA- GA	PCA- Bag
1	5.5	2	5.5	9.5	9.5	7.5	4	12	1	11	7.5	3
2	11	2	10	9	7.5	6	3.5	12	1	7.5	5	3.5
3	6	2.5	7	10	11	4.5	9	12	1	4.5	8	2.5
4	8	3	6.5	9	11.5	6.5	4	10	1	5	11.5	2
5	9	5.5	7.5	10	12	7.5	4	11	1	5.5	3	2
Aver.	7.9	3.0	7.3	9.5	10.3	6.4	4.9	11.4	1.0	6.7	7.0	2.6

In the procedure of the Nemenyi test, the critical distance (CD), in contrast to the distances of average ranks among various classifiers, is calculated. This can be represented as^53^:

6 $${\mathrm{CD}} = q_{\alpha ,\infty ,k}\sqrt {\frac{{k\left( {k + 1} \right)}}{{12\,N}}}$$

where the critical value ($q_{\alpha ,\infty ,k}$) is based on the studentized-range statistic that is tabulated in standard statistical textbooks^50^. It is obvious that the critical distance is determined by the number of models (*k*), the number of data sets (*D*), and the significance level (*α*).

In Fig. 8d, we present the critical diagram of all classifiers for 5-fold cross-validation data sets using Nemenyi’s post hoc test at the significance level of 0.1. The average ranks of the models are drawn on the horizontal axis; thus, the best ranking method is on the left-most side of the diagram. The specific rank of the 12 approaches is recorded in Table 3. The critical distance (*CD*) is represented by a series of lines traversing the average ranks in Fig. 8d and is calculated to be 6.9094.

The various algorithms are distinguished by color, the average rank of each algorithm is represented by points, and the critical distance is represented by horizontal lines. Drawing a dashed auxiliary line from the end of the better ranking algorithm to the horizontal axis, we can judge whether there is overlap among the algorithms. The intersecting lines mean that the models cannot be clearly distinguished. In this paper, more attention is focused on the performance differences of the algorithms combined with PCA and SDAE.

As shown in Fig. 8d, only the distance of the AdaBoost decision tree exceeds the critical distance (marked with a black tick). This means that SDAE dimension reduction is more useful for improving the performance of the AdaBoost decision tree model than PCA. However, for other approaches, the critical distances are not exceeded. Therefore, we cannot conclude that there is a significant difference between the algorithms with various dimension reduction methods.

**Fig. 9:**
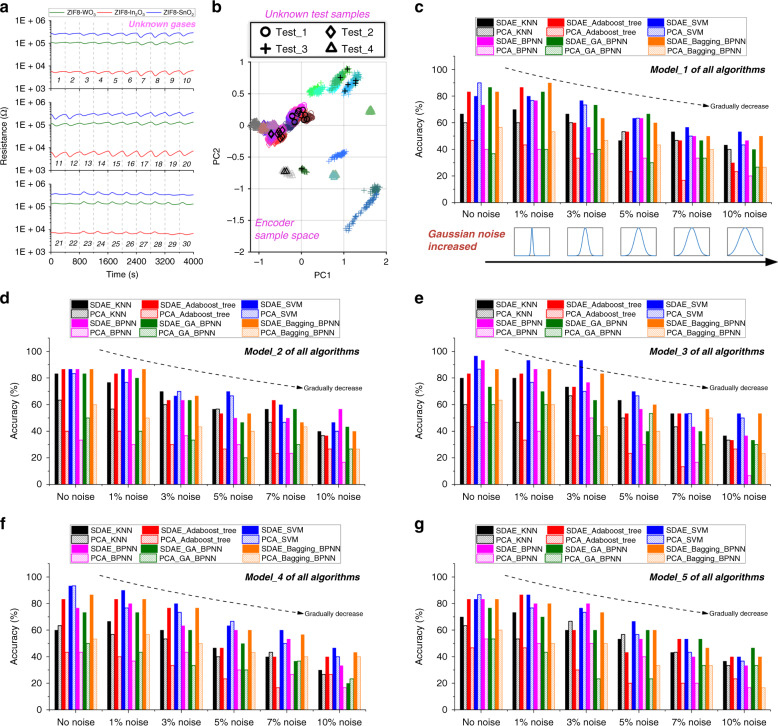
Recognition results of the unknown gases. **a** The original signals of the GS microchip upon exposure to 30 unknown gases. **b** SDAE-reduced unknown samples mapped in PCA space. For the unknown data set with noise impact, the classification accuracy of various as-trained models on the basis of **c** the first, **d** the second, **e** the third, **f** the fourth, and **g** the fifth cross-validation data set

For the as-trained model, noise disturbance in new data sets may cause it to produce completely incorrect predictions^30^. To further assess the generalization ability of various models, a series of additional experiments marked as the unknown data set are carried out, as presented in Supplementary Table S3. Then, the as-trained recognition models are applied to infer the components of an unknown gas. Since the experiment for an unknown data set is obtained in another period, the signal drift might result in a deviation from the original data set. The signals of the GS microchip upon exposure to 30 unknown target gases are shown in Fig. 9a. Taking the third cross-validation data set as an example, the dimensionality of the unknown initial sample can be reduced from 18 to 5 via SDAE. Then, the unknown reduced samples are mapped to the PCA space with black marks. In Fig. 9b, different shapes represent different test groups, the same as those in Supplementary Table S1. It can be observed that the unknown samples’ clustering regions in PCA space have a correlation with the test group.

To further explore the antinoise abilities of the SDAE-based and PCA-based models, Gaussian noise with different amplitudes is artificially added to the unknown data set:

7 $${\mathrm{noise}} \sim N\left( {\mu ,\,\sigma ^2} \right)$$

where the mean *μ* is set to 0. The standard deviation *σ* is a ratio of the unknown data set and determines the noise amplitude. For the unknown data set with noise impact, Fig. 9c–g presents a comparison of the classification accuracies of the various as-trained models from the 5 cross-validation data sets; additionally, the antinoise ability of each model can be observed (the noise amplitude gradually increases from left to right). The average results are summarized in Table 4, where the recognition accuracy of each model gradually decreases with increasing noise. This proves that noise has an impact on the recognition accuracy of the model. Note that regardless of the magnitude of the noise, the recognition accuracy of the SDAE-based models is higher than that of the PCA-based models. The accuracy of some models is nearly twice as high. This means that the deep-level features extracted by SDAE can more efficiently represent the original sample, and the as-trained model also has a stronger antinoise level and generalization ability than PCA. Additionally, for the bagging-BPNN, the improvement in the accuracy due to integrating several sub-learners is verified again.

**Table 4:** The average accuracies of the different algorithms under SDAE and PCA dimension reduction on unknown data sets with varying amounts of noise.

Algorithm	Average accuracy (%)					
	No noise	1% noise	3% noise	5% noise	7% noise	10% noise
SDAE-KNN	72.00	73.33	66.00	53.33	49.33	37.33
PCA-KNN	62.00	54.67	61.33	51.33	44.67	34.00
SDAE-AdaBoost tree	84.00	84.67	66.67	50.00	51.33	36.00
PCA-AdaBoost tree	44.00	40.67	32.67	23.33	18.00	25.33
SDAE-SVM	88.00	87.33	78.67	66.67	56.67	48.00
PCA-SVM	88.00	76.67	72.00	64.00	48.67	42.00
SDAE-BPNN	82.67	82.00	68.00	56.67	47.33	41.33
PCA-BPNN	43.33	39.33	43.33	32.67	24.00	15.33
SDAE-GA-BPNN	78.67	75.33	64.00	52.67	46.67	36.67
PCA-GA-BPNN	50.00	45.33	33.33	31.33	32.67	28.00
SDAE-Bagging-BPNN	85.33	85.33	72.67	58.67	51.33	45.33
PCA-Bagging-BPNN	58.67	54.00	46.67	40.00	41.33	26.67

With the specific classification accuracies for unknown data sets with varying amounts of noise in Table 4, we continue to employ the Friedman and Nemenyi tests to evaluate whether the overall performance of these 12 recognition learners is statistically significant. First, 12 algorithms for various data sets are ranked in Supplementary Table S4. Then, the corrected Friedman statistic is used to verify the hypothesis of whether all algorithms exhibit the same performance. According to Eqs. (4) and (5), $\Gamma _F$ can be calculated to be 69.072, which is larger than the critical value of the *F* distribution (1.968) at the significance level of 0.05. Therefore, the hypothesis that all algorithms exhibit the same performance is false.

**Fig. 10:**
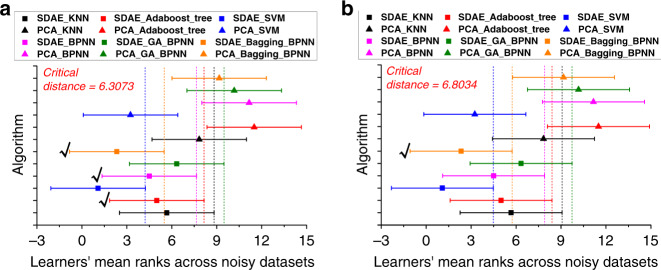
Hypothesis test for the unknown data sets with noise. The results of the Nemenyi post hoc test, where **a** *α* is equal to 0.1 and **b** *α* is equal to 0.05

To further assess the performance of 12 classifiers for unknown data sets with various noise, the Nemenyi test is applied. At the significance levels of 0.1 and 0.05, the critical distances are calculated to be 6.3073 and 6.8034, respectively. The mean ranks of the 12 approaches are summarized in Supplementary Table S4. For the significance level of 0.1 (Fig. 10a), the models (the AdaBoost decision tree, BPNN, and bagging-BPNN) using SDAE for dimensionality reduction exhibit better performance than those using PCA (marked with a black tick) because the distances between each SDAE-based and PCA-based model exceed the critical distance. However, for other algorithms with various dimension reductions, their critical distances exhibit overlap, so significant differences cannot be assumed. Furthermore, in Fig. 10b, even if we decrease the significance to 0.05, the remaining bagging-BPNN still shows that SDAE exhibits better performance than PCA in statistics.

In Supplementary S3, the influence of humidity on the GS microchip is investigated. A series of experiments on binary mixtures of SO_2_F_2_ and SO_2_ under different humidities are also performed. The results show that the bagging-BPNN based on SDAE achieves the highest classification accuracy.

Moreover, to verify the broad application of the proposed method, we perform additional experiments to investigate whether the GS sensor can quantify gas mixtures with large differences in concentrations, as illustrated in Supplementary S4. Four less toxic gases (CH_4_, H_2_, C_2_H_2_, and CO) in very different concentrations are chosen as the measured gases, with calibration concentrations of 500, 10, 250, and 25 ppm, respectively. Apart from the gas sources, the test system and recognition algorithms are the same as those in previous discussions. The results show that even when four gases are mixed, the GS microchip still has the ability to recognize them. Experiments with orders of magnitude differences in gas concentration also show that the GS microchip can quantify gas mixtures with very different concentrations.

## Conclusions

We fabricated a GS microchip loaded with three gas-sensitive materials. A portable gas detection system was built around the GS microchip to identify multicomponent SF_6_ decomposition products. Using SDAE dimensionality reduction, the original data sets with 18 dimensions could be reduced to 5 high-level features. Six machine learning algorithms were employed to successfully identify 47 gas mixtures. To evaluate the generalization ability, 30 groups of unknown gases with various artificial noise were assigned. Regardless of the magnitude of the added noise, the SDAE-based models exhibited better performances than the PCA-based models. Finally, hypothesis testing indicated at 95% confidence that the bagging-BPNN with the SDAE method exhibits superior performance. To verify the broad application of the proposed procedure, we chose another four gases (CH_4_, H_2_, C_2_H_2_, and CO) at calibration concentrations of 500, 10, 250, and 25 ppm, respectively. The experimental results showed that the GS sensor could quantify gas mixtures with very different concentrations.

## Material and methods

### Materials and gases

All reagents used in this experiment were analytically pure without further purification and purchased from Sinopharm Chemical Reagent Co., Ltd. (Shanghai, China). Calibration gases (H_2_S, SO_2_F_2_, SOF_2_, and SO_2_) of ~100 ppm were stored in steel cylinders purchased from the National Institute of Metrology, China.

### Fabrication process of GS microchip

The GS microchip was fabricated on a one-side polished p-type silicon wafer (4 inches in diameter, 525 μm in thickness). All processing of metal patterns was achieved by photolithography. A specific schematic of the micromachining process is depicted in Supplementary Fig. S1. (1) A 200-nm-thick Si_3_N_4_ layer was deposited on the silicon wafer by low-pressure chemical vapor deposition (LPCVD). (2) Using a photolithography technique to customize patterns, a 30/200 nm Cr/Pt layer was deposited on the Si_3_N_4_ layer by an E-beam evaporator to serve as a microheater. According to the area and square resistance of the Pt electrodes, the designed resistance of the heater was ~47 Ω. (3) A 30/400/30 nm Al_2_O_3_/Si_3_N_4_/Al_2_O_3_ thin film was used as an electrical insulating layer between the microheater and test electrodes. The insulating layer was deposited through plasma-enhanced chemical vapor deposition (PECVD). To expose the Pt pad for wire bonding, a reactive ion etching (RIE) method was utilized to etch the upper insulating layer. (4) The test electrodes for loading gas-sensing materials had a width of 20 μm with a gap size of 20 μm. Through the lift-off technique, a 30/250 nm Cr/Au thin film was patterned to make the pair of electrodes and wire bonding pads. (5) Square windows were opened on the silicon wafer backside by deep inductive coupled plasma (ICP) etching to achieve thermal isolation of the chip and reduced heat loss.

### Production of MOF-based metal oxide sensing materials

Metal-organic frameworks (MOFs) have received extensive attention due to their high surface area, ultrahigh porosity, and tunable structures. Zinc-based zeolite imidazole framework (ZIF8) is a widely reported MOF catalyst. Electrospinning can effectively synthesize 1D nanowires. With this method, ZIF8 was tightly anchored to nanowires by electrospinning. Following fast calcination, ZIF8 can be transformed into ZnO. Heterojunctions generated between ZnO catalysts and metal oxide nanotubes can improve gas-sensing properties by modulating the depletion layers. The specific synthesis process of ZIF8 and three gas-sensing composites are described in the Electronic Supplementary Material (ESM).

### Assembly of the portable device

All modules were encapsulated in a box sized 22 × 14 × 13 cm. A gas chamber <150 mL was made of nylon 12, which has superior corrosion resistance. The GS microchip could be placed in this chamber, where an electrical feed-through and a gas inlet and outlet were also designed. Supplementary Fig. S3 shows the main circuit board. STM32F103C8T6 was chosen as the MCU of this board. This was enough to undertake the task of data acquisition and transmission. LCDs and serial ports were used in the communication methods with the users. Using a serial interface, the data could be transmitted to the computer. The response signals of the GS microchip could also be recorded for further analysis. After testing was finished, the rinsing pump was turned on, and the gas chamber was flushed with ambient air through a one-way valve.

### Characterization

The structure of the as-fabricated chip was documented using atomic force microscopy (AFM, NX10), with a medium and stiffer middle silicon probe (OMCL-AC160TS) with a nominal radius of 10 nm. The distribution of the surface temperature of the device was monitored by an IR camera (Fluke Ti95). The morphology and nanostructure of the gas-sensitive materials were examined by field emission scanning electron microscopy (FESEM, Zeiss Gemini SEM 500).

## Supplementary information

Supplementary Information
