# Supplementary material for: Multicomponent SF6 decomposition product sensing with a gas-sensing microchip
Source: Microsyst Nanoeng. 2021 Mar 1;7:18. doi: 10.1038/s41378-021-00246-1 (PMC8433328; doi:10.1038/s41378-021-00246-1)
Supplement: Supplementary file 1 — Supplementary Information [file 41378_2021_246_MOESM1_ESM.doc]

Supplementary information

for

**Multicomponent SF_6_ decomposition product sensing with a gas-sensing microchip**

Jifeng Chu^§^, Aijun Yang^§^, Qiongyuan Wang, Xu Yang, Dawei Wang, Xiaohua Wang*, Huan Yuan, and Mingzhe Rong

*State Key Laboratory of Electrical Insulation and Power Equipment, Xi'an Jiaotong University, Xi'an 710049, China.*

**Correspondence:* Xiaohua Wang ([xhw@mail.xjtu.edu.cn](mailto:xhw@mail.xjtu.edu.cn))

*^§^ Contribute equally to this work.*

This file includes:

**S1. Fabrication of GS microchip and measurement system**

S1.1 Synthesis of sensing materials

S1.2 Fabrication of GS microchip

S1.3 Measurement system

**S2. Feature extraction and pattern recognition**

S2.1 Feature matrix from the signals

S2.2 SDAE processing for extracting deep features

S2.3 Schematic of machine learning algorithms

S2.4 Cross-validated results of various test sets

**S3. Mixed gas experiment under different humidity**

**S4. Mixed gas experiment with great difference in concentration**

**S1 Fabrication of GS microchip and measurement system**

S1.1 Synthesis of sensing materials

**(1) Synthesis of ZIF8.** 0.293 g of Zinc nitrate hexahydrate ([Zn(NO_3_)_2_·6H_2_O]) and 0.6489 g of 2-methylimidazole (Hmin, 99.0%) were dissolved in 20 mL methanol, separately. At room temperature, a solution of Hmin was rapidly added into Zn(NO_3_)_2_·6H_2_O solution under magnetic stirring for 1 h. After that, the milky white dispersion was purified by centrifugation and washing with ethanol for several times. The final products were dried at 50 °C in air.

**(2) Synthesis of** **ZIF8-WO_3_**. 0.4 g of ammonium metatungstate hydrate (AMH, [(NH_4_)_6_H_2_W_12_O_40_·H_2_O]) and 0.5 g of Polyvinylpyrrolidone (PVP, molecular weight of∼1300000 g/mol, K90) were dissolved in 3 mL deionized (DI) water for electrospinning. 40 mg ZIF8 was dispersed in 1 mL DI water to generate suspension, and adding it to the electrospinning solution with continuously stirred for 4 h. At 25 °C with 30% RH, a syringe pump injected the as-prepared solution at a feeding rate of 0.3 mm/min. During electrospinning, high voltage (16 kV) and distance (15 cm) were maintained between the needle tip and the stainless-steel foil collector. Finally, the produced films were calcinated at 500 °C for 1h in air with a heating rate of 5 °C/min.

**(3) Synthesis of** **ZIF8-In_2_O_3_**. 25 mg ZIF8 was dispersed in 10 mL DMF. Then, 0.5 g ([In(NO_3_)_3_·4.5H_2_O]) and 1.288g PVP (K90) were added into above solution with stirring for 4 h until the viscous solution formed. After thoroughly stirring, the electrospinning solution was transferred to the syringe. At 25 °C with 30% RH, the syringe pump injected at a feeding rate of 0.3 mm/min. High voltage (16 kV) and distance (15 cm) were maintained between the needle tip and the stainless-steel foil collector. Finally, the produced films were calcinated at 500 °C for 1h in air with a heating rate of 5 °C/min.

**(4) Synthesis of** **ZIF8-SnO_2_**. 0.25 g of tin (II) chloride dihydrate (SnCl_2_·2H_2_O) and 0.35 g of PVP (K90) were dissolved in the mixed solution of 2 mL ethanol and 1.5mL DMF. Then, 17 mg ZIF8 was added to the mixed solution with continuously stirred for 4 h. At 25 °C with 30% RH, a syringe pump injected the as-prepared solution at a feeding rate of 0.25 mm/min. High voltage (16 kV) and distance (15 cm) were maintained between the needle tip and the stainless-steel foil collector. Finally, the produced films were calcinated at 600 °C for 1h in air with a heating rate of 5 °C/min.

S1.2 Fabrication process of GS microchip


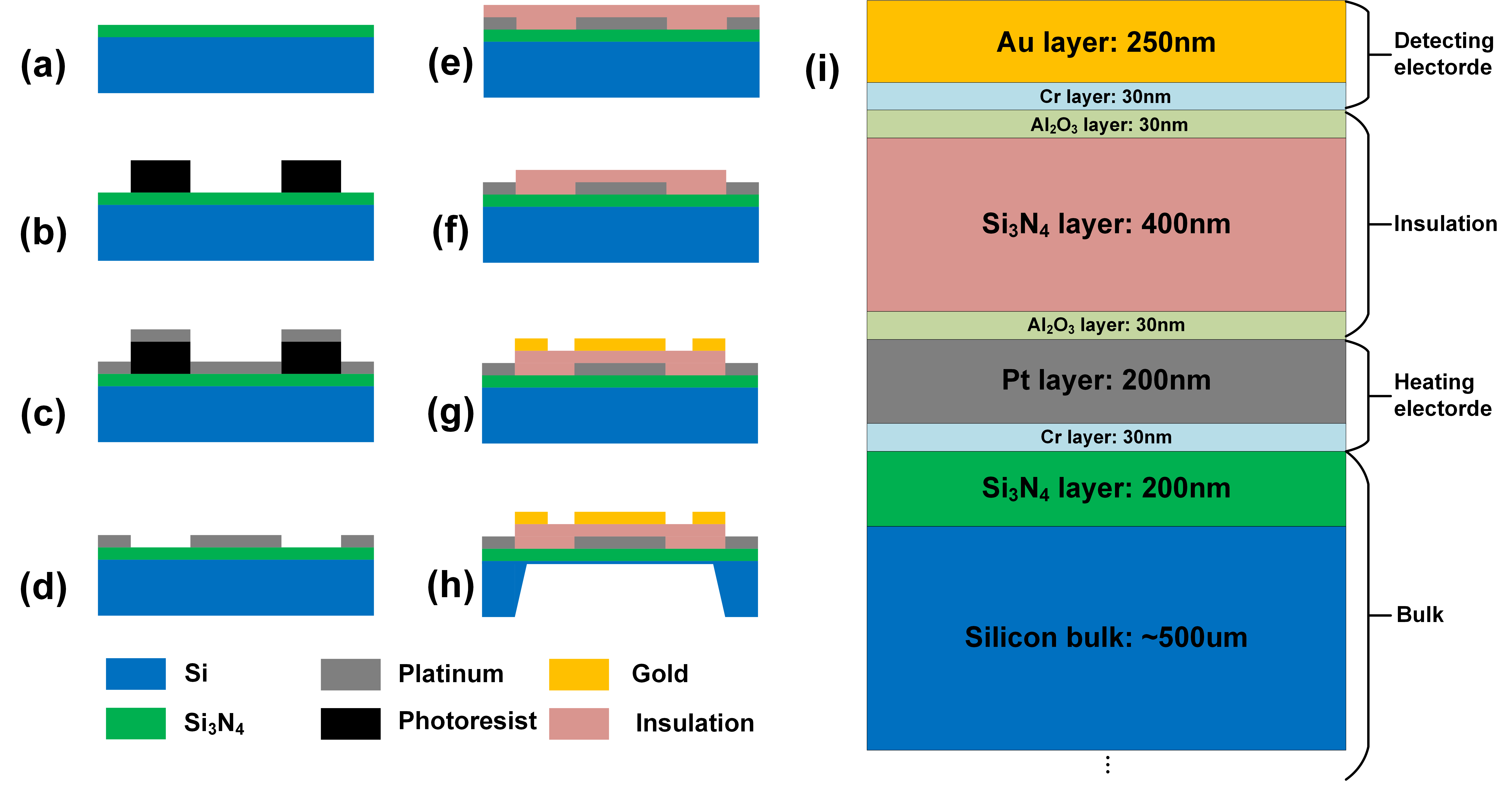


**Fig. S1** (a)-(h) The fabrication process of the micro gas-sensing chip. (i) Cross section of the GS microchip.


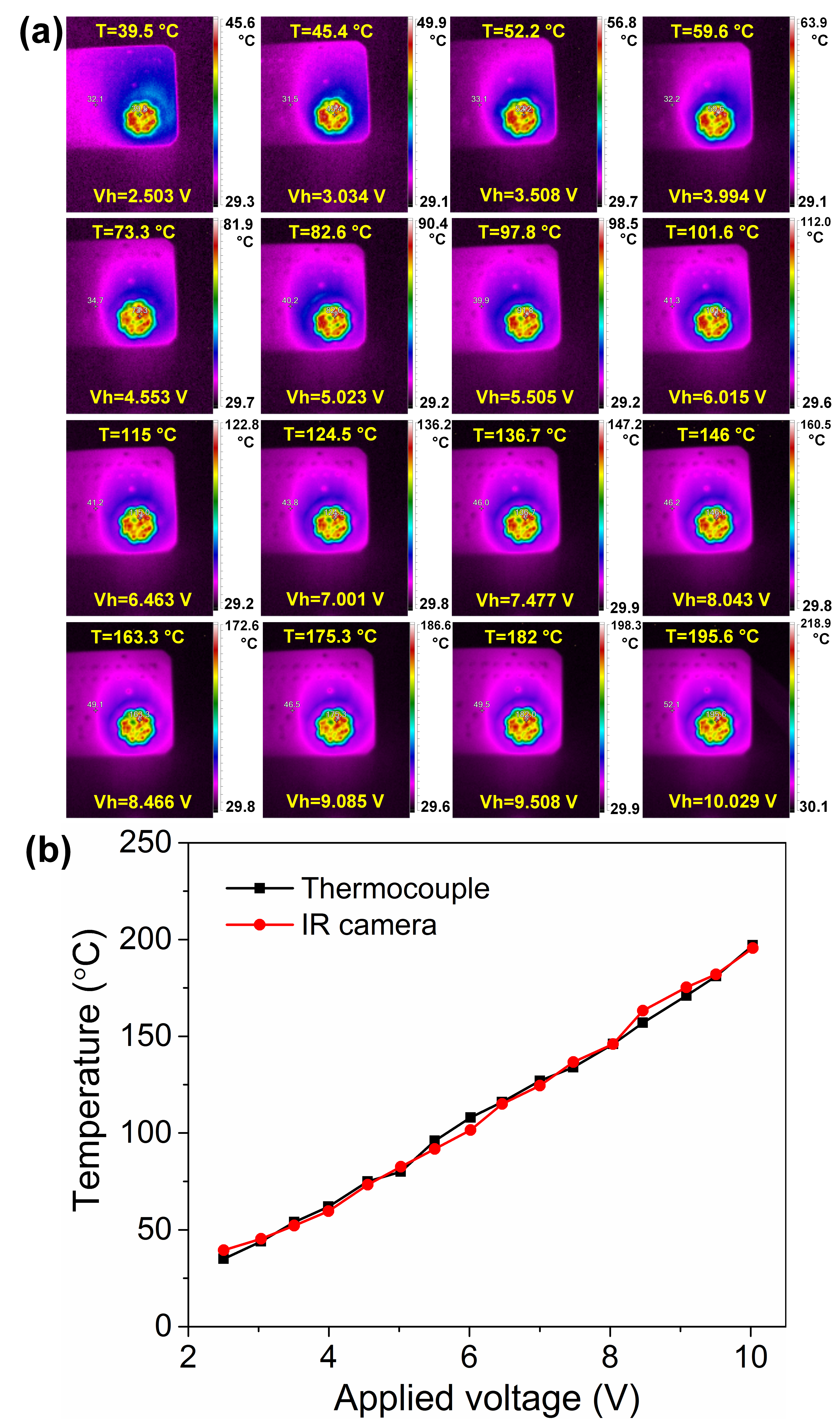


**Fig. S2** (a) Temperature images recorded by the IR camera at different heating voltages. (b) The curves of temperature versus applied voltage jointly calibrated by IR camera and thermocouple.

S1.3 Measurement system


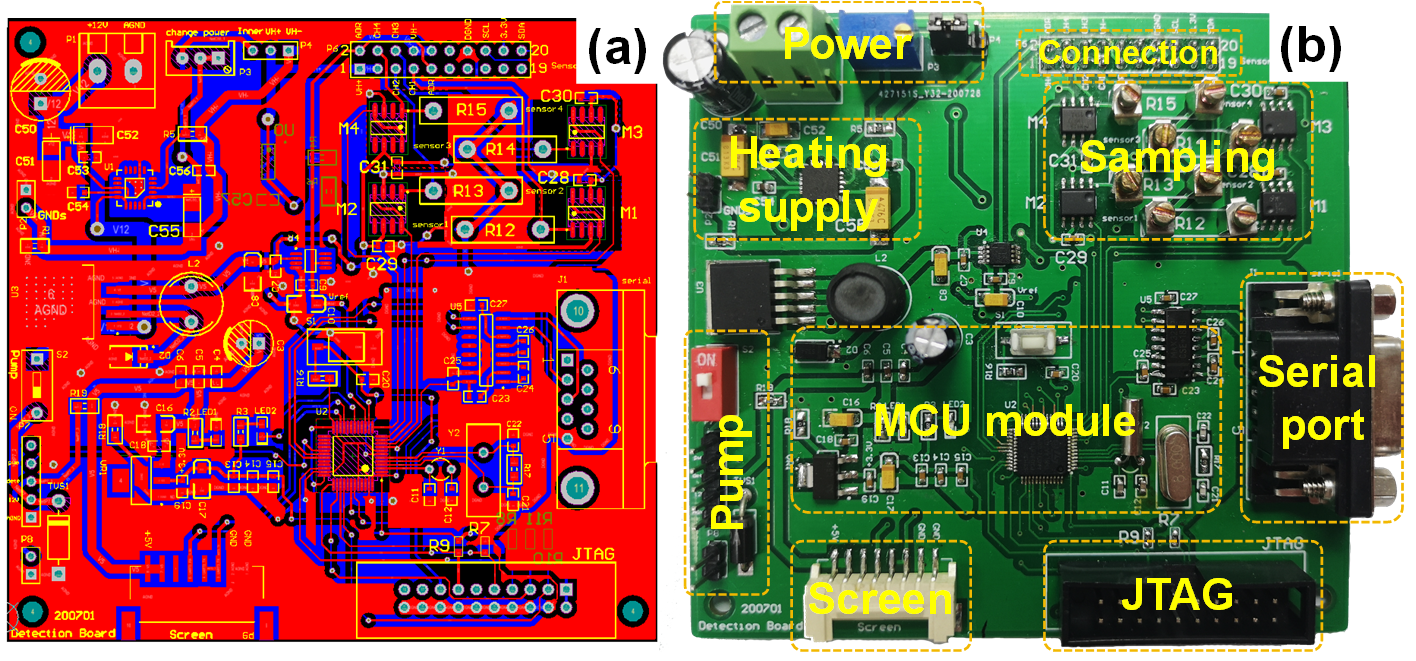


**Fig. S3** Practical picture of main circuit board.


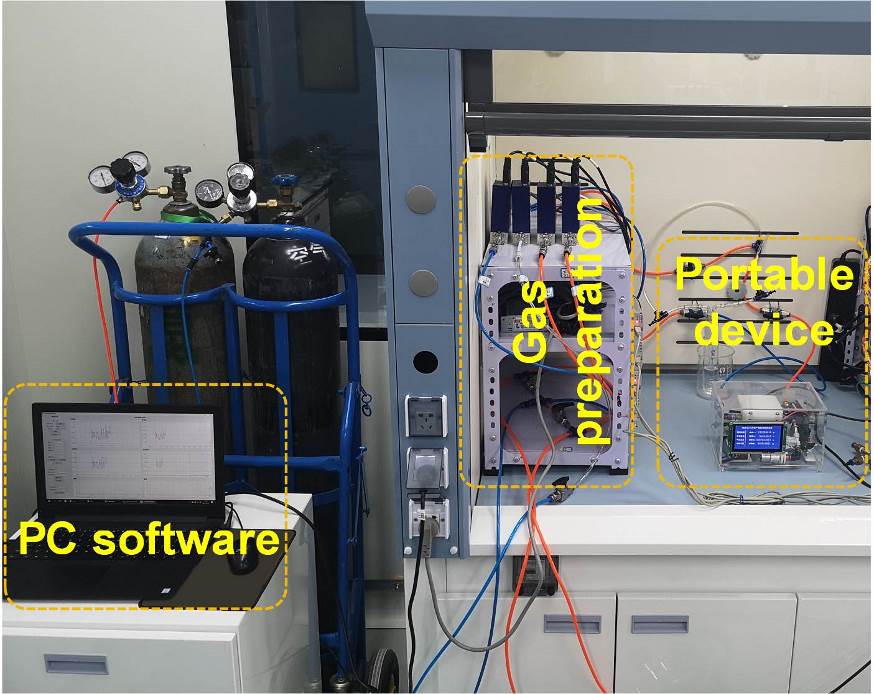


**Fig. S4** Physical diagram of measurement system.

**Table S1 Gas mixing schemes of four SF_6_ decomposition components.**

| **Experiment** | **Group** | **Label** | **H_2_S (ppm)** | **SO_2_F_2_ (ppm)** | **SOF_2_ (ppm)** | **SO_2_ (ppm)** |
| --- | --- | --- | --- | --- | --- | --- |
| **SO_2_F_2_, SO_2_** | Test 1 | 1 | 0 | 10 | 0 | 0 |
|  |  | 2 | 0 | 0 | 0 | 10 |
|  |  | 3 | 0 | 10 | 0 | 10 |
|  |  | 4 | 0 | 30 | 0 | 0 |
|  |  | 5 | 0 | 0 | 0 | 30 |
|  |  | 6 | 0 | 30 | 0 | 30 |
|  |  | 7 | 0 | 50 | 0 | 0 |
|  |  | 8 | 0 | 0 | 0 | 50 |
|  |  | 9 | 0 | 50 | 0 | 50 |
|  |  | 10 | 0 | 30 | 0 | 10 |
|  |  | 11 | 0 | 10 | 0 | 30 |
|  |  | 12 | 0 | 50 | 0 | 10 |
|  |  | 13 | 0 | 10 | 0 | 50 |
|  |  | 14 | 0 | 50 | 0 | 30 |
|  |  | 15 | 0 | 30 | 0 | 50 |
| **H_2_S, SO_2_** | Test 2 | 16 | 10 | 0 | 0 | 0 |
|  |  | 17 | 10 | 0 | 0 | 10 |
|  |  | 18 | 30 | 0 | 0 | 0 |
|  |  | 19 | 30 | 0 | 0 | 30 |
|  |  | 20 | 50 | 0 | 0 | 0 |
|  |  | 21 | 50 | 0 | 0 | 50 |
|  |  | 22 | 30 | 0 | 0 | 10 |
|  |  | 23 | 10 | 0 | 0 | 30 |
|  |  | 24 | 50 | 0 | 0 | 10 |
|  |  | 25 | 10 | 0 | 0 | 50 |
|  |  | 26 | 50 | 0 | 0 | 30 |
|  |  | 27 | 30 | 0 | 0 | 50 |
| **SOF_2_, SO_2_** | Test 3 | 28 | 0 | 0 | 10 | 0 |
|  |  | 29 | 0 | 0 | 10 | 10 |
|  |  | 30 | 0 | 0 | 30 | 0 |
|  |  | 31 | 0 | 0 | 30 | 30 |
|  |  | 32 | 0 | 0 | 50 | 0 |
|  |  | 33 | 0 | 0 | 50 | 50 |
|  |  | 34 | 0 | 0 | 30 | 10 |
|  |  | 35 | 0 | 0 | 10 | 30 |
|  |  | 36 | 0 | 0 | 50 | 10 |
|  |  | 37 | 0 | 0 | 10 | 50 |
|  |  | 38 | 0 | 0 | 50 | 30 |
|  |  | 39 | 0 | 0 | 30 | 50 |
| **SO_2_F_2_, SOF_2_, SO_2_** | Test 4 | 40 | 0 | 10 | 10 | 10 |
|  |  | 41 | 0 | 10 | 10 | 30 |
|  |  | 42 | 0 | 30 | 10 | 10 |
|  |  | 43 | 0 | 10 | 30 | 10 |
|  |  | 44 | 0 | 30 | 10 | 30 |
|  |  | 45 | 0 | 30 | 30 | 10 |
|  |  | 46 | 0 | 10 | 30 | 30 |
|  |  | 47 | 0 | 30 | 30 | 30 |

**S2. Feature extraction and pattern recognition**

S2.1 Feature matrix from the signals


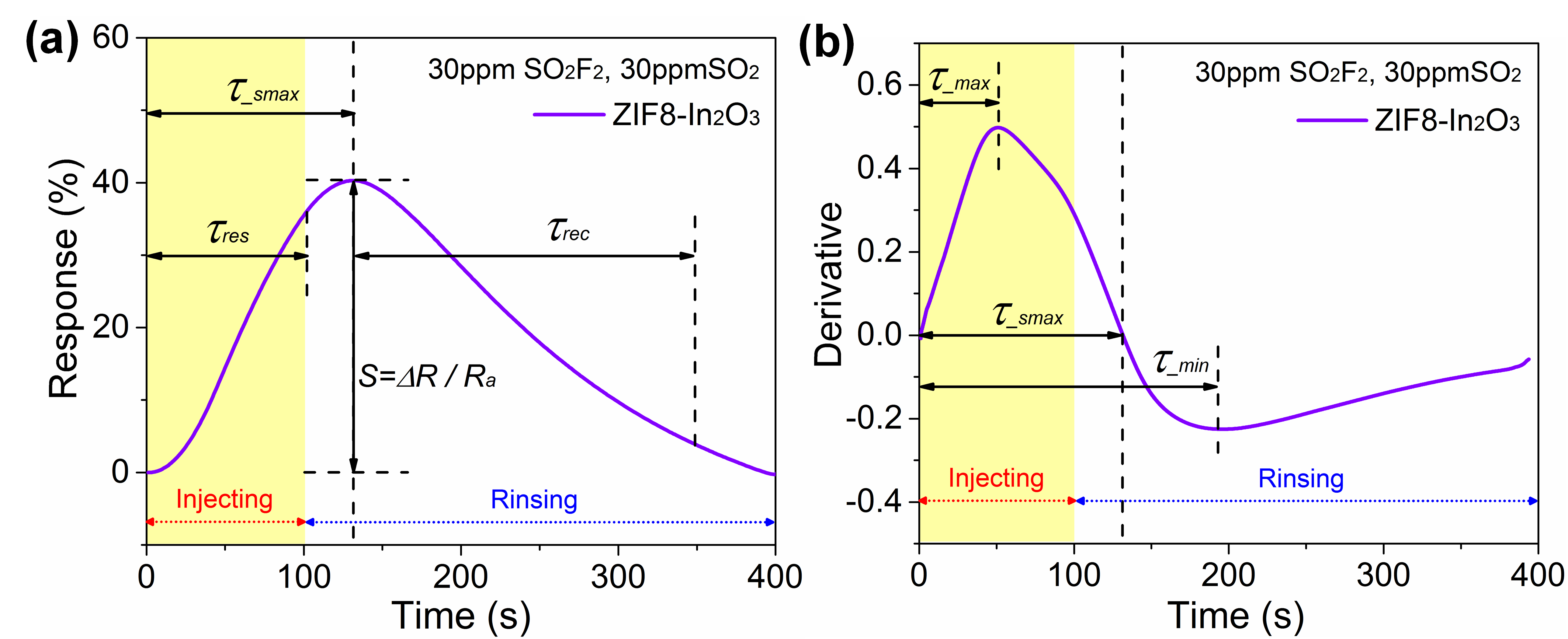


**Fig. S5** Taken ZIF8-In_2_O_3_ as an example, six features extracted from the GS microchip’s signal. (a) Primitive response-recovery curve. (b) First derivative curve.


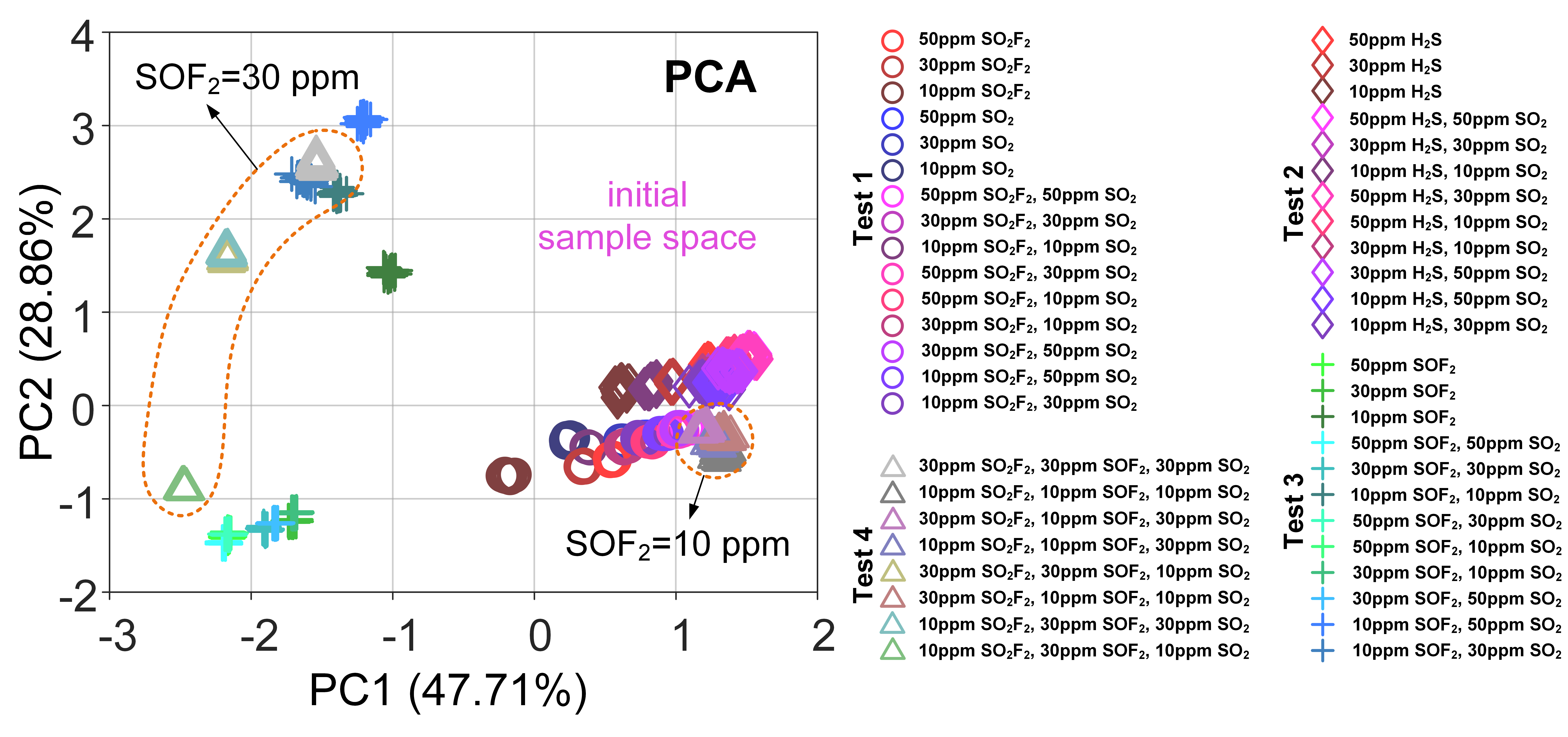


**Fig. S6** Samples’ visualization in two-dimension space by PCA.

S2.2 SDAE processing for extracting deep features


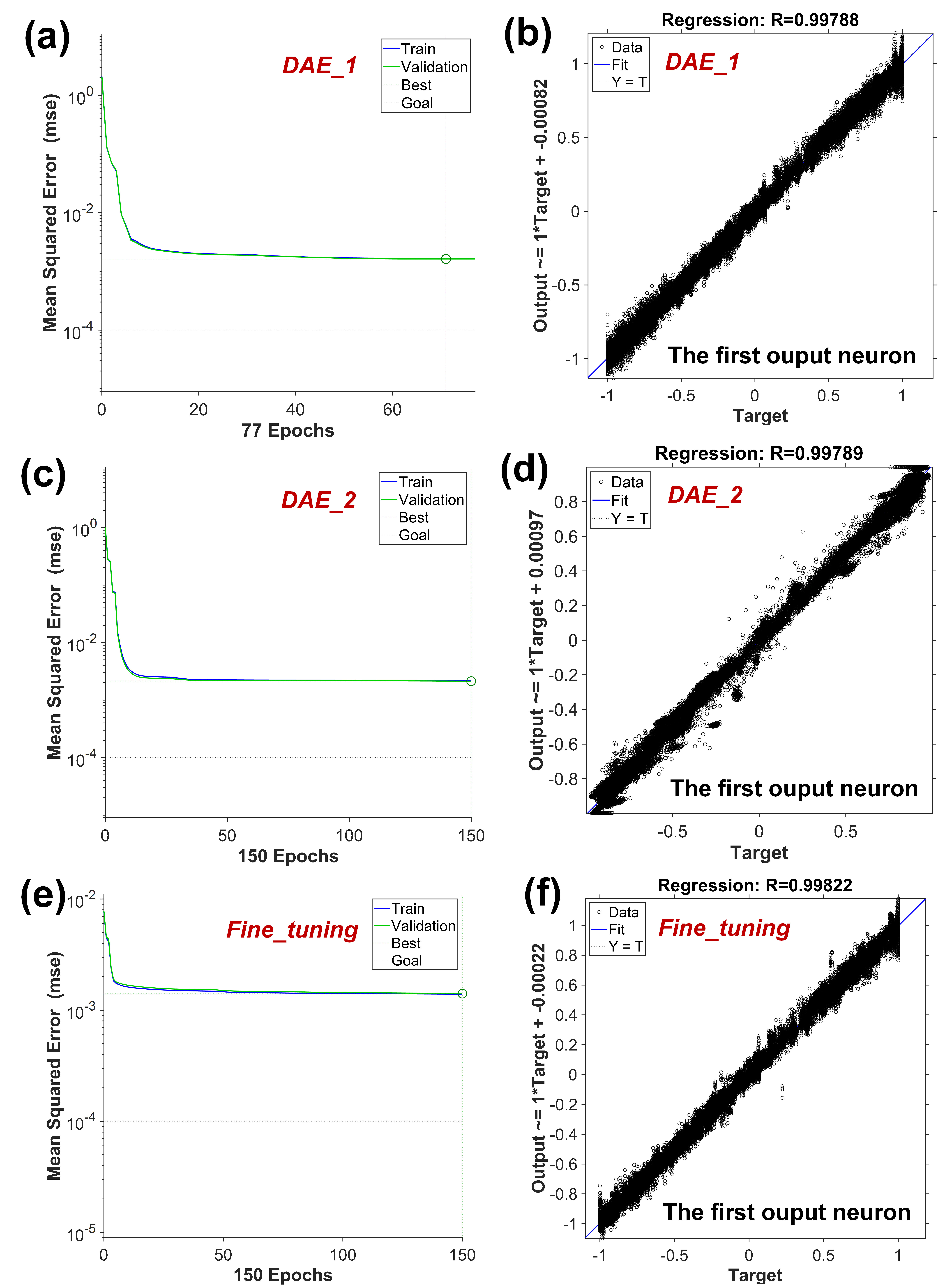


**Fig. S7** The SDAE training curves for (a) DAE_1, (c) DAE_2, (e) fine_tuning. The error regression curves of the first neuron in output layer, for (b) DAE_1, (d) DAE_2, (f) fine_tuning.

S2.3 Schematic of machine learning algorithms


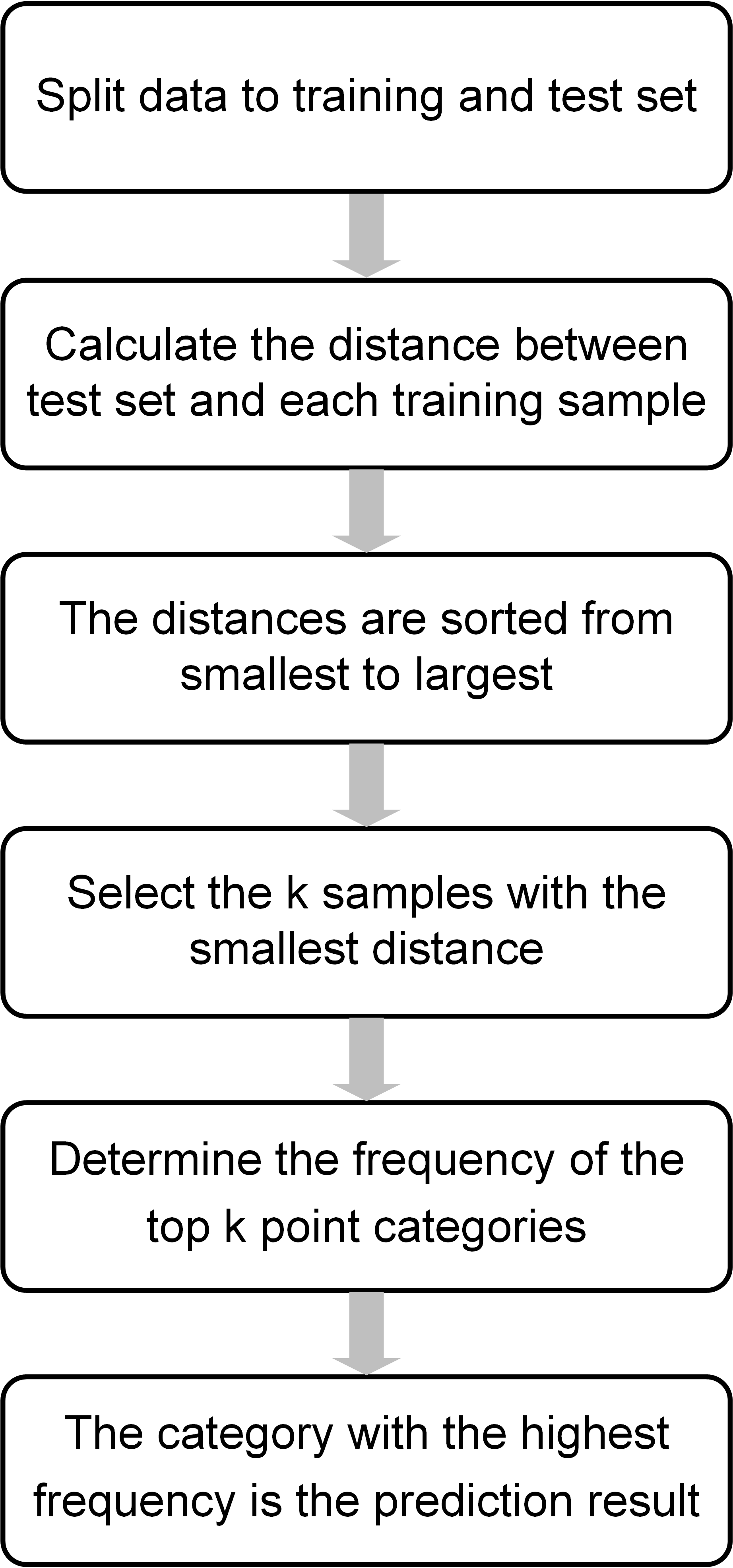


**Fig. S8** The schematic of KNN.


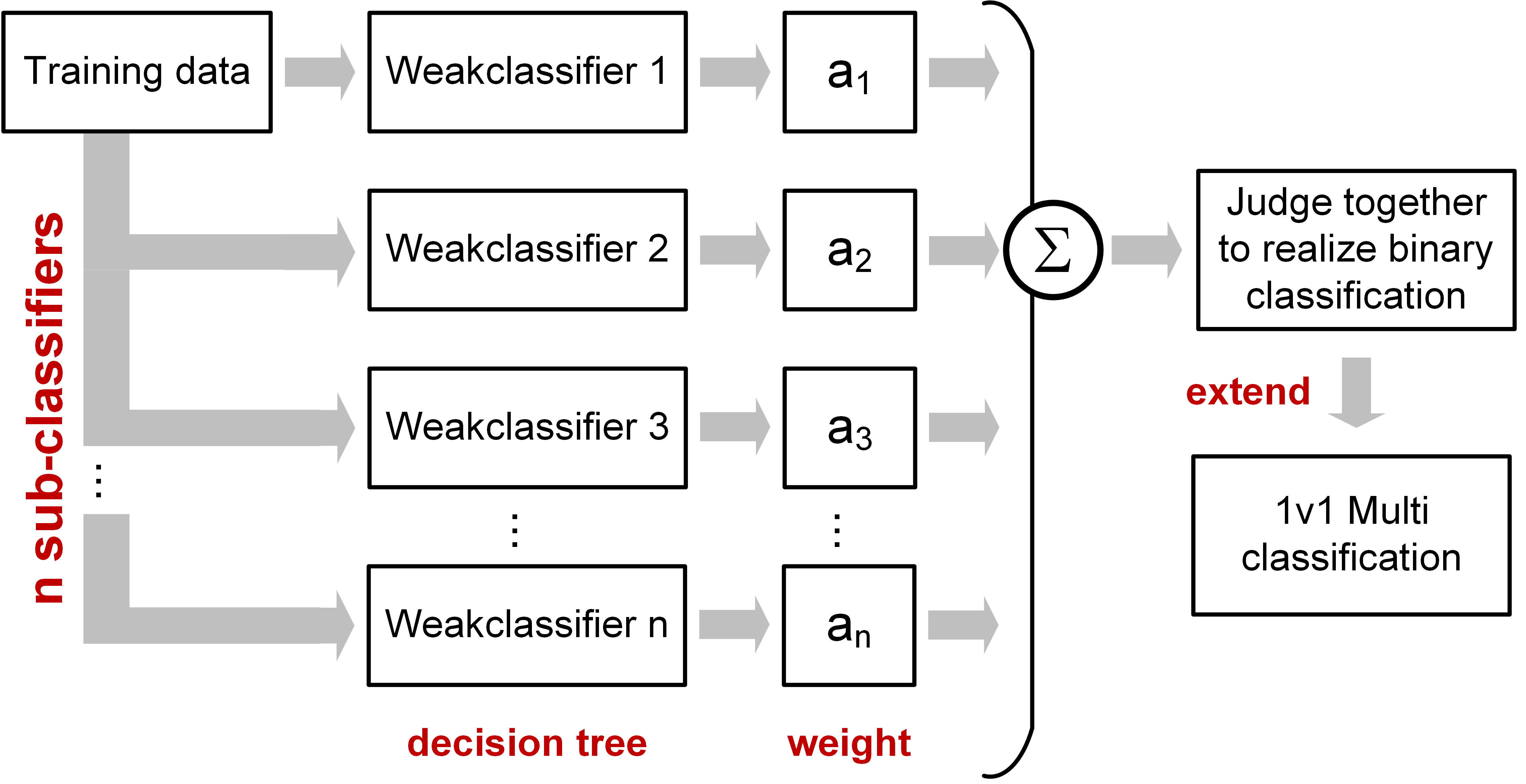


**Fig. S9** The schematic of AdaBoost decision tree.


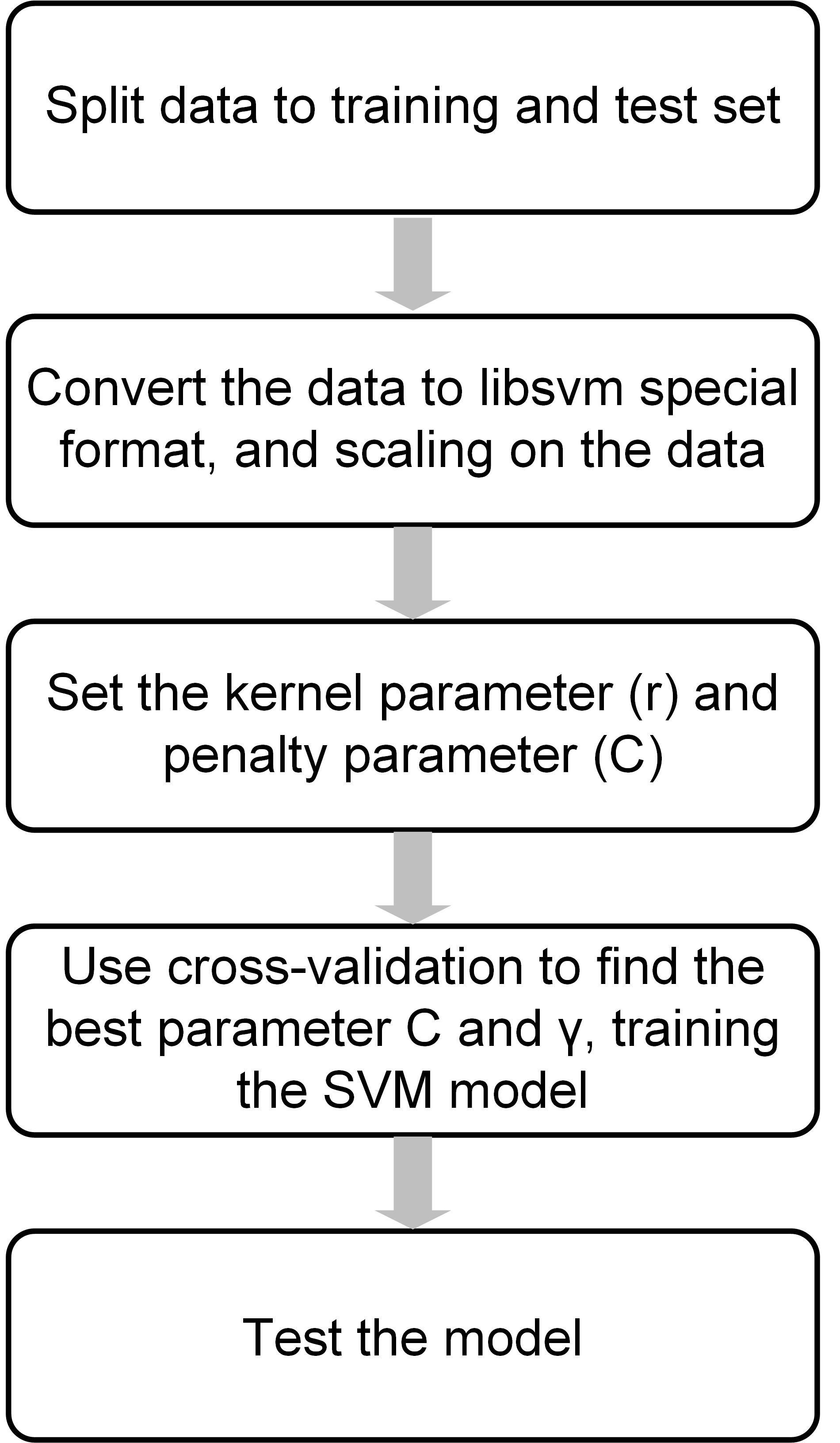


**Fig. S10** The schematic of SVM.


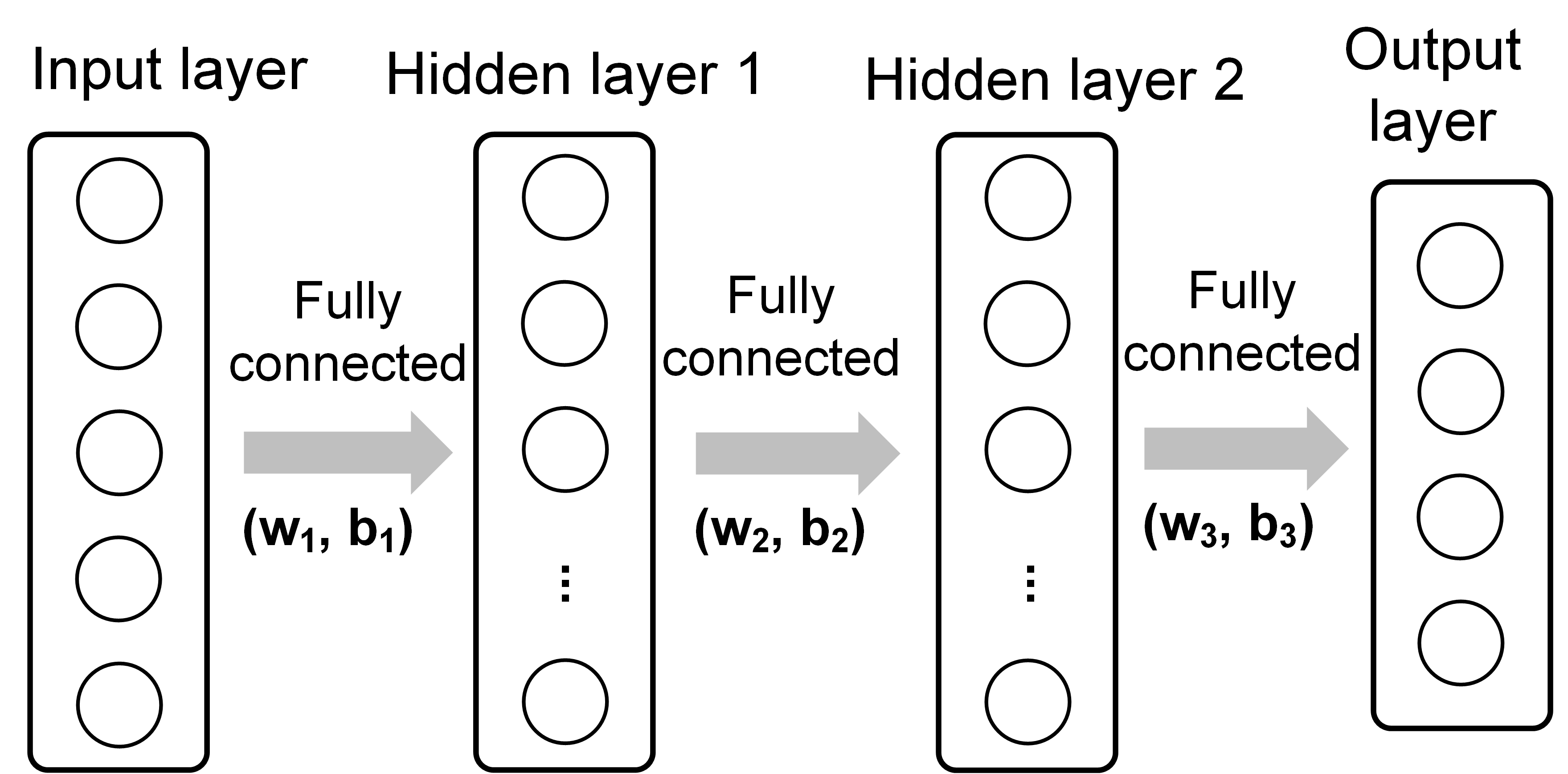


**Fig. S11** The schematic of BPNN.


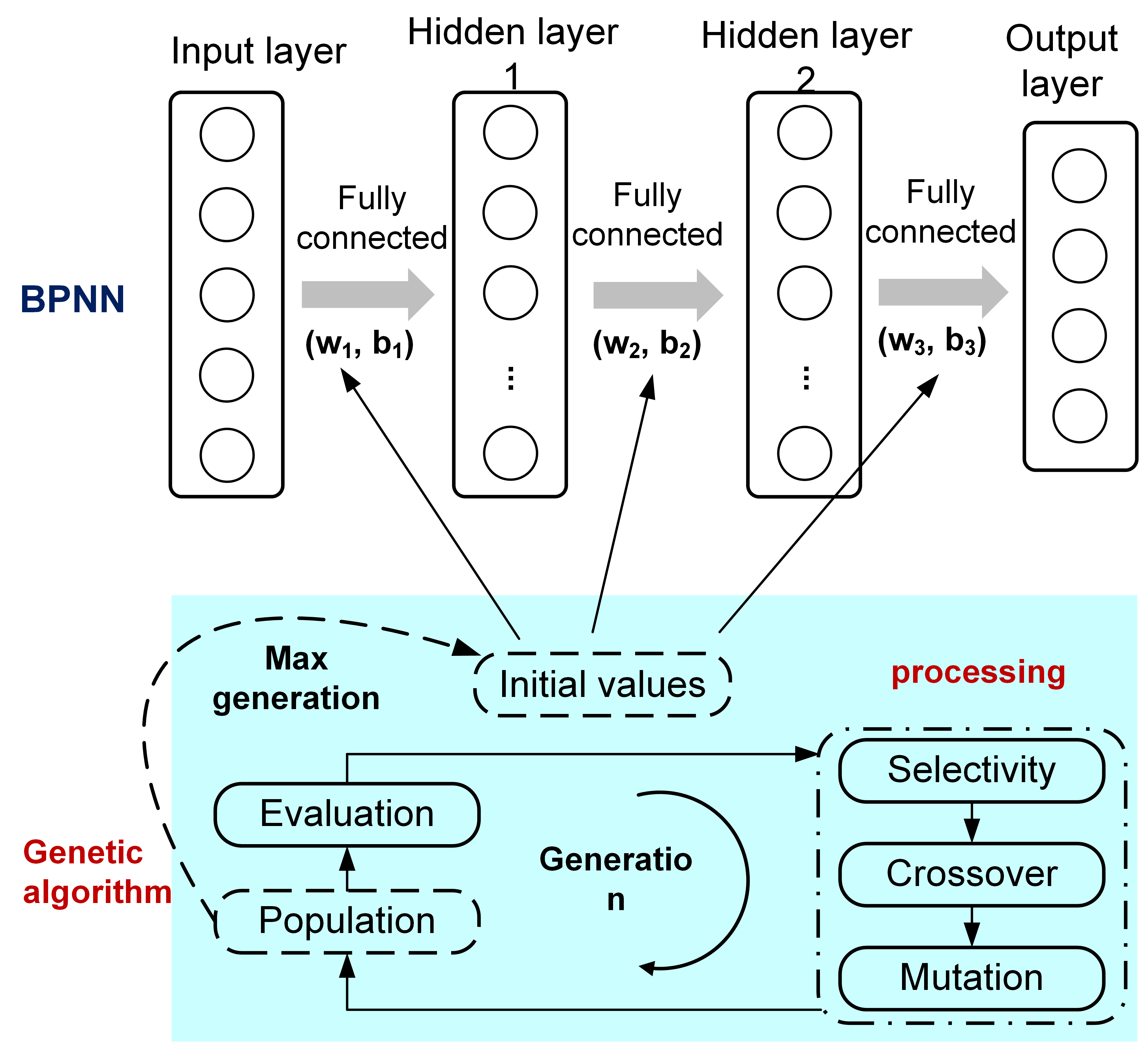


**Fig. S12** The schematic of GA-BPNN.


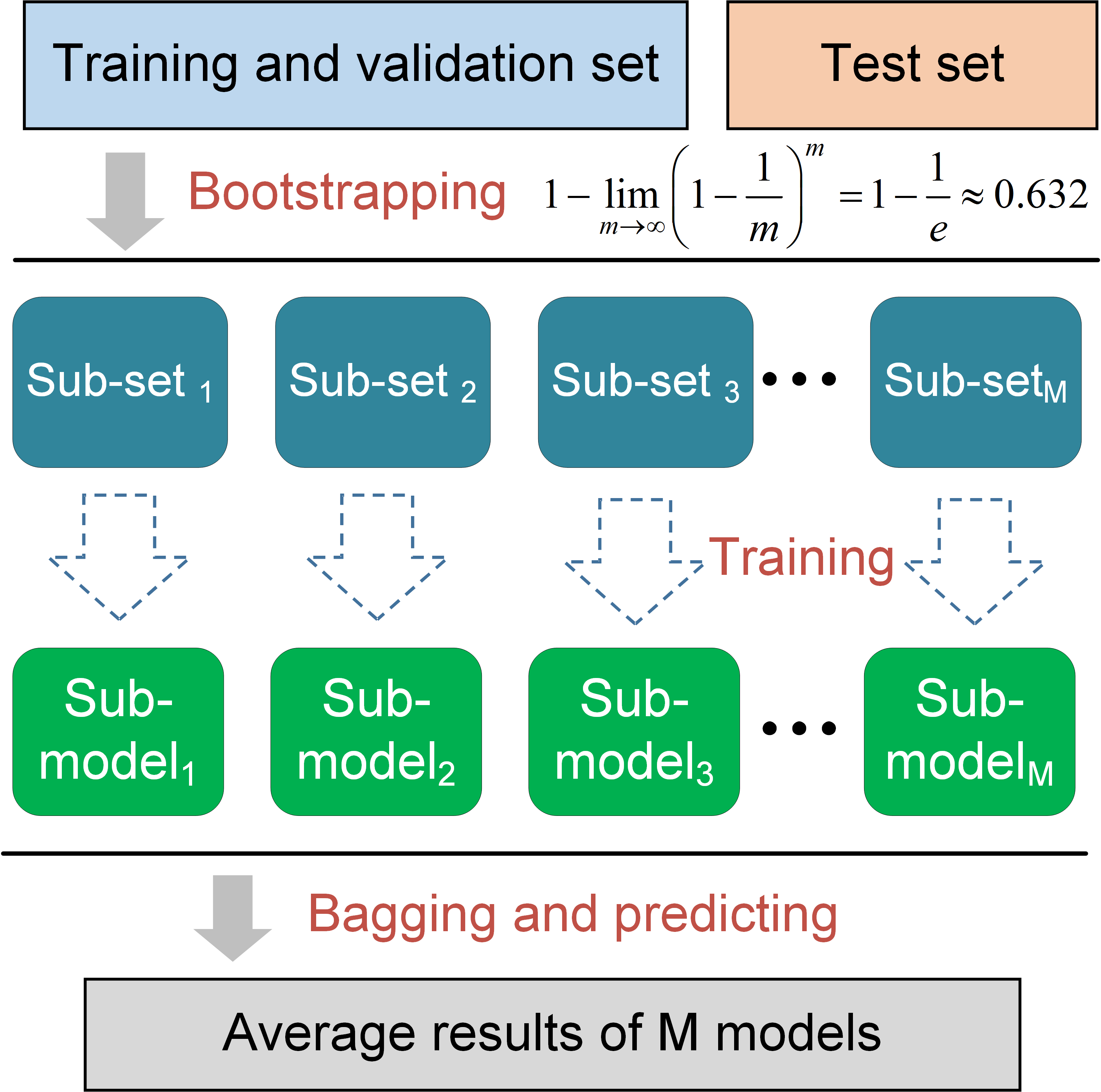


**Fig. S13** The schematic of Bagging-BPNN.

S2.4 Cross- validated results of various test sets

**Fig. S14** (a) The flowchart for screening the number of hidden layers and neurons. (b-f) Under various hidden layers and neurons, the recognition errors based on cross-validated models.


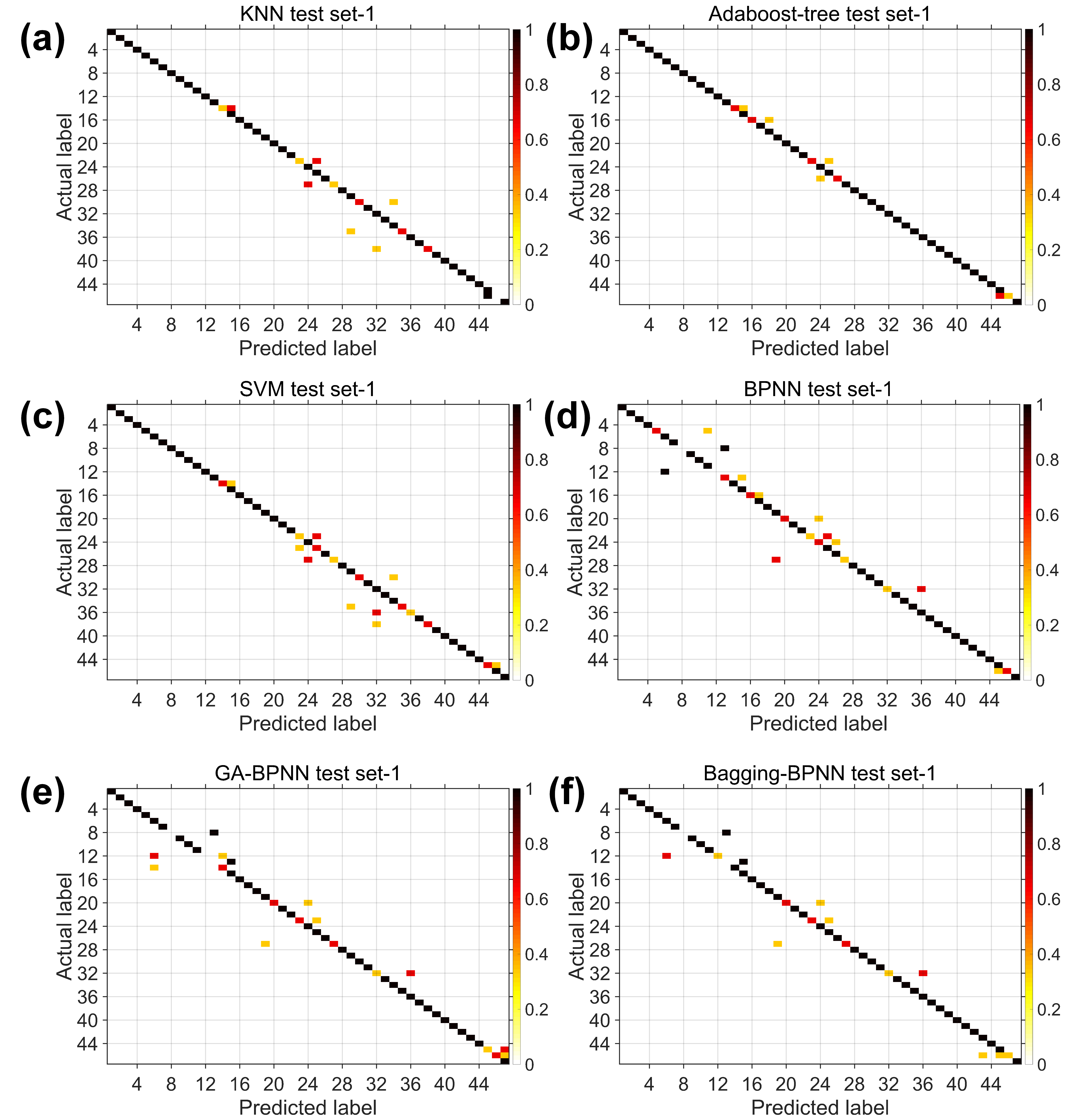


**Fig. S15** For the first cross-validation dataset, the confusion matrix of (a) KNN, (b) AdaBoost decision tree, (c) SVM, (d) BPNN, (e) GA-BPNN, (f) Bagging-BPNN.


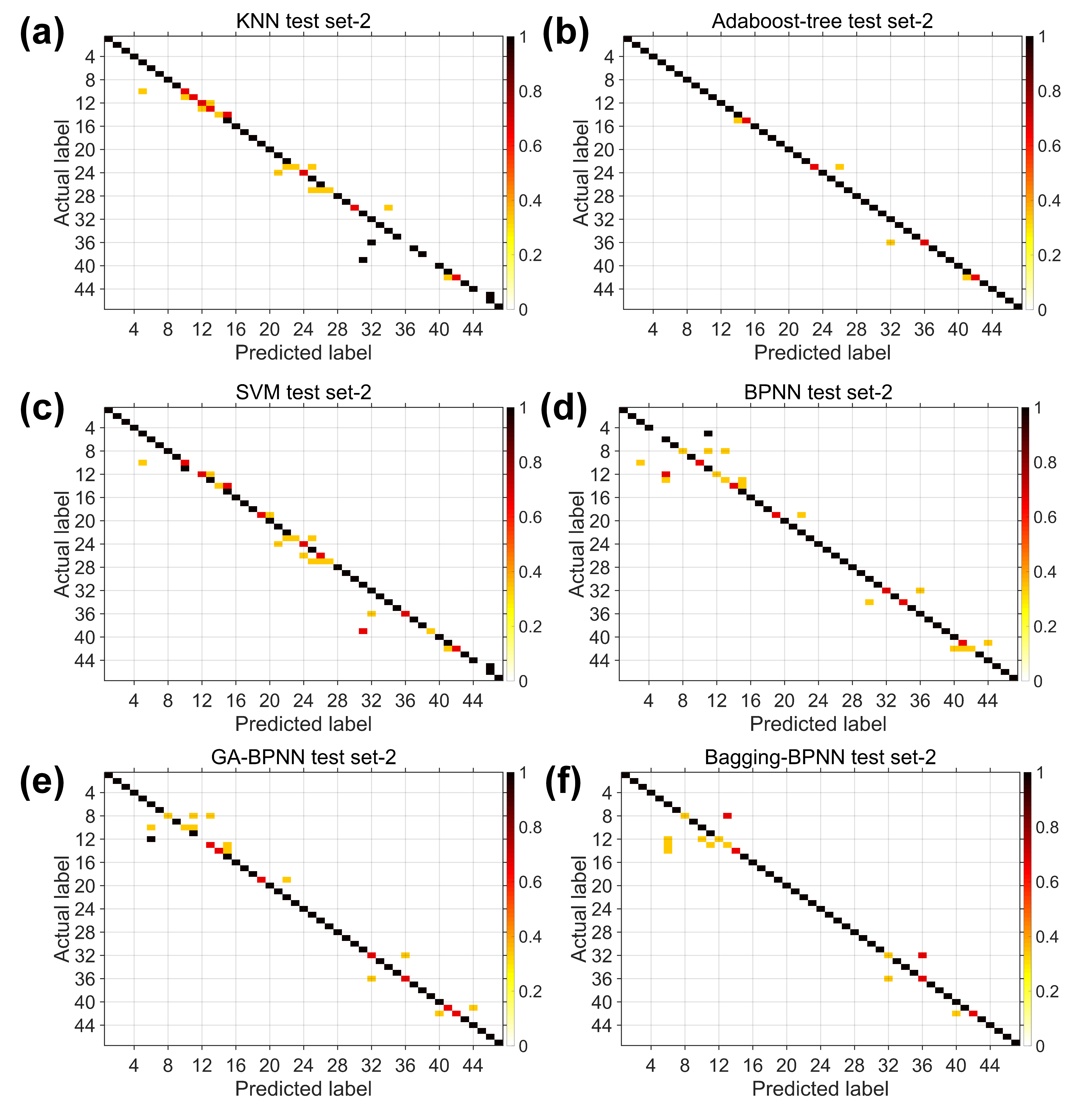


**Fig. S16** For the second cross-validation dataset, the confusion matrix of (a) KNN, (b) AdaBoost decision tree, (c) SVM, (d) BPNN, (e) GA-BPNN, (f) Bagging-BPNN.


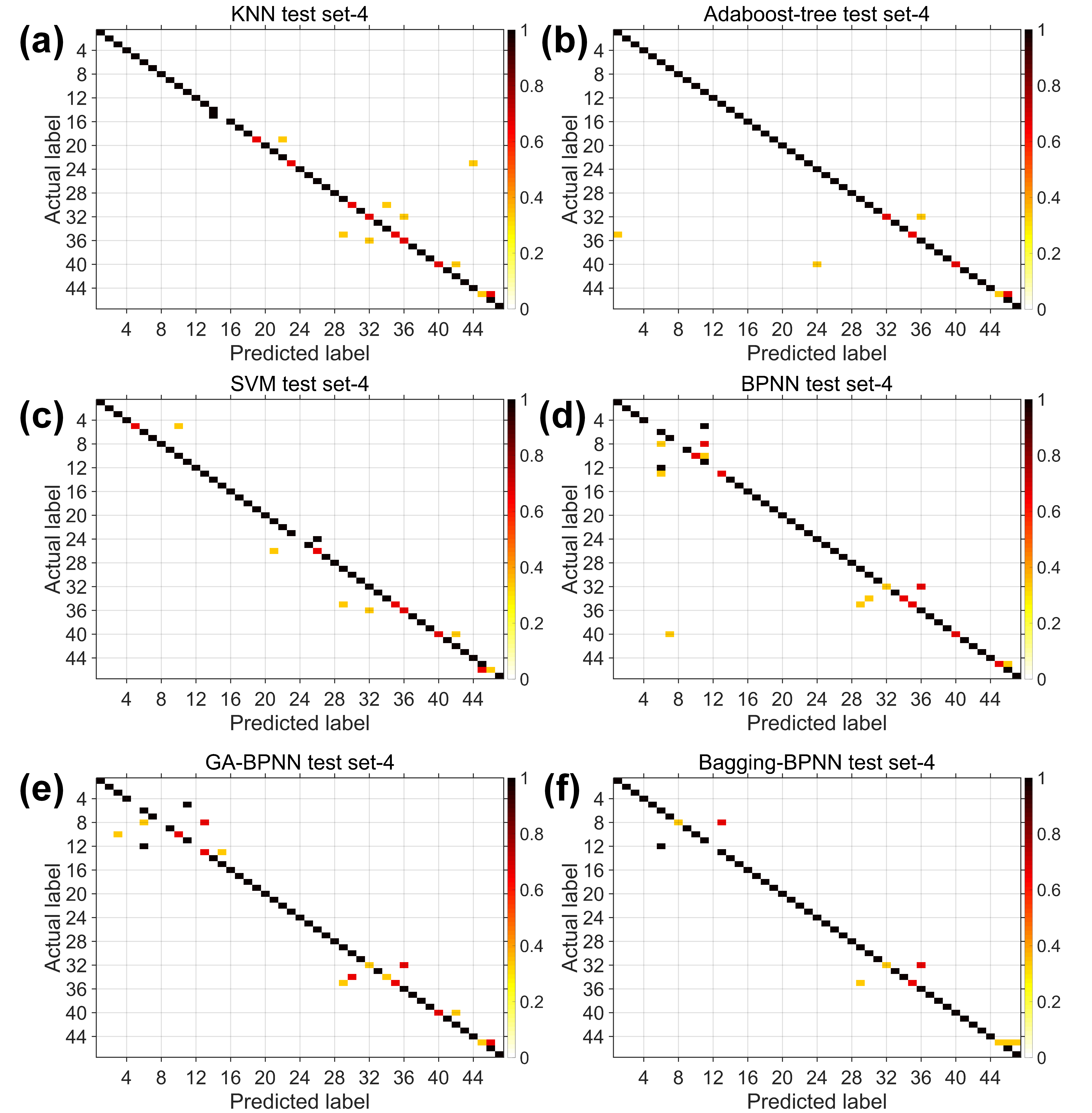


**Fig. S17** For the fourth cross-validation dataset, the confusion matrix of (a) KNN, (b) AdaBoost decision tree, (c) SVM, (d) BPNN, (e) GA-BPNN, (f) Bagging-BPNN.


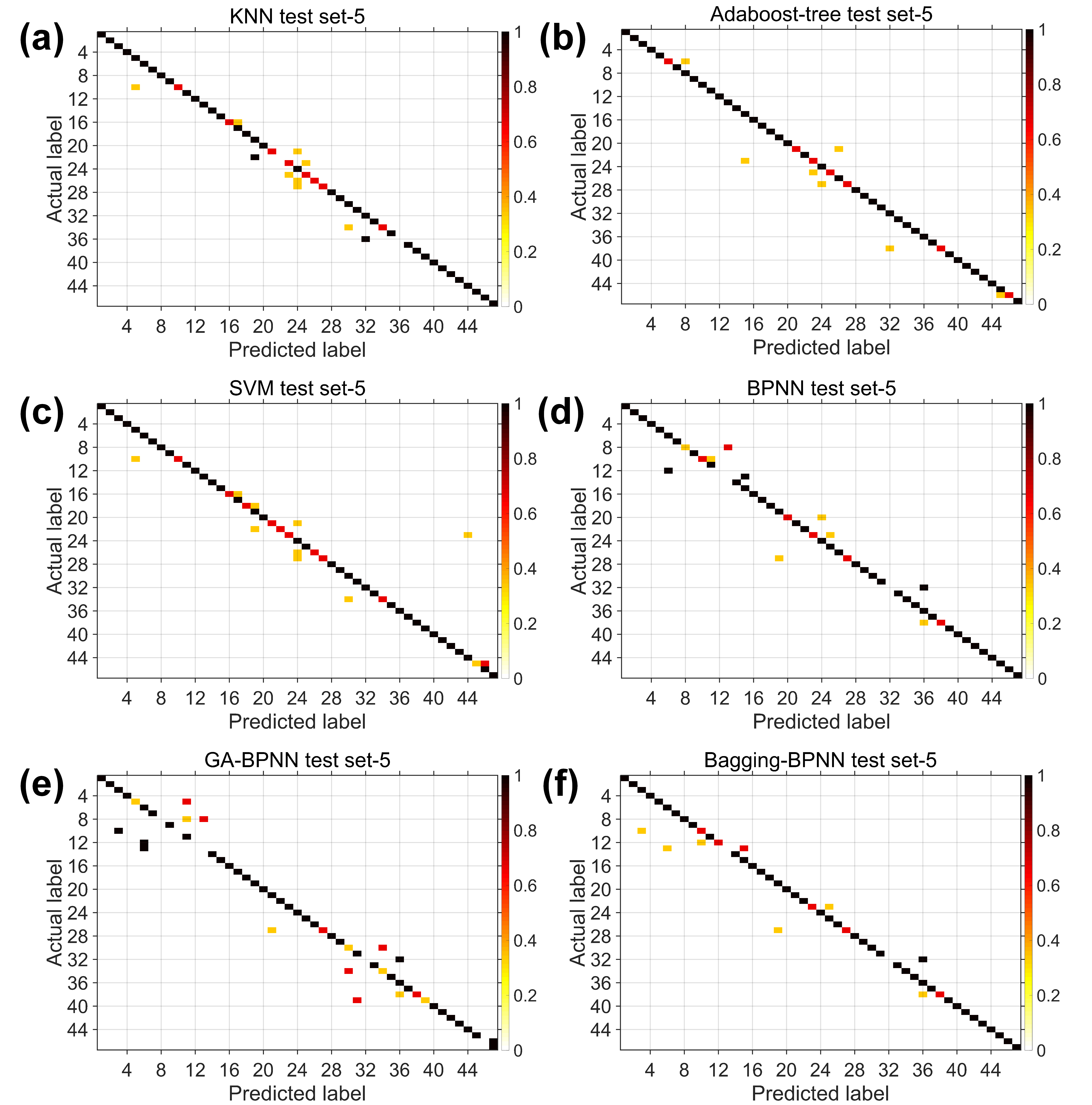


**Fig. S18** For the fifth cross-validation dataset, the confusion matrix of (a) KNN, (b) AdaBoost decision tree, (c) SVM, (d) BPNN, (e) GA-BPNN, (f) Bagging-BPNN.

**Table S2** For the third cross-validation dataset, the regression prediction results by Bagging-BPNN based on SDAE dimensionality reduction.

| **Number of test set** | **Predicted result** | | | | **Actual result** | | | |
| --- | --- | --- | --- | --- | --- | --- | --- | --- |
|  | H_2_S (ppm) | SO_2_F_2_ (ppm) | SOF_2_ (ppm) | SO_2_ (ppm) | H_2_S (ppm) | SO_2_F_2_ (ppm) | SOF_2_ (ppm) | SO_2_ (ppm) |
| **1** | 0.01 | 9.69 | 0.82 | -0.19 | 0 | 10 | 0 | 0 |
| **2** | 0.00 | 10.03 | 0.63 | -0.39 | 0 | 10 | 0 | 0 |
| **3** | -0.01 | 9.54 | 0.73 | 0.11 | 0 | 10 | 0 | 0 |
| **4** | 0.39 | 1.39 | 0.50 | 10.95 | 0 | 0 | 0 | 10 |
| **5** | 0.41 | -0.68 | 0.94 | 11.04 | 0 | 0 | 0 | 10 |
| **6** | 0.33 | -0.38 | 0.46 | 11.50 | 0 | 0 | 0 | 10 |
| **7** | 0.07 | 9.33 | -0.16 | 10.82 | 0 | 10 | 0 | 10 |
| **8** | 0.08 | 9.54 | -0.14 | 10.23 | 0 | 10 | 0 | 10 |
| **9** | 0.01 | 11.47 | -0.14 | 10.43 | 0 | 10 | 0 | 10 |
| **10** | 0.02 | 30.89 | -0.13 | -0.38 | 0 | 30 | 0 | 0 |
| **11** | -0.01 | 32.35 | -0.10 | -0.76 | 0 | 30 | 0 | 0 |
| **12** | -0.04 | 33.09 | -0.07 | -0.87 | 0 | 30 | 0 | 0 |
| **13** | 0.06 | 4.72 | -0.33 | 27.73 | 0 | 0 | 0 | 30 |
| **14** | 0.02 | 4.31 | -0.37 | 28.84 | 0 | 0 | 0 | 30 |
| **15** | 0.02 | 4.02 | -0.36 | 29.14 | 0 | 0 | 0 | 30 |
| **16** | -0.14 | 33.11 | -0.60 | 29.94 | 0 | 30 | 0 | 30 |
| **17** | -0.13 | 33.33 | -0.62 | 30.64 | 0 | 30 | 0 | 30 |
| **18** | -0.06 | 30.72 | -0.74 | 30.83 | 0 | 30 | 0 | 30 |
| **19** | -0.13 | 47.36 | 0.28 | -0.27 | 0 | 50 | 0 | 0 |
| **20** | -0.06 | 45.57 | 0.13 | 1.14 | 0 | 50 | 0 | 0 |
| **21** | -0.09 | 46.68 | 0.23 | -0.85 | 0 | 50 | 0 | 0 |
| **22** | -0.14 | 4.77 | -0.74 | 44.54 | 0 | 0 | 0 | 50 |
| **23** | -0.08 | 0.19 | -0.75 | 48.43 | 0 | 0 | 0 | 50 |
| **24** | -0.18 | 4.29 | -0.76 | 46.74 | 0 | 0 | 0 | 50 |
| **25** | -0.15 | 48.61 | 0.22 | 49.22 | 0 | 50 | 0 | 50 |
| **26** | 0.09 | 49.92 | 0.35 | 50.03 | 0 | 50 | 0 | 50 |
| **27** | 0.07 | 49.82 | 0.33 | 49.76 | 0 | 50 | 0 | 50 |
| **28** | 0.02 | 26.15 | 0.24 | 13.23 | 0 | 30 | 0 | 10 |
| **29** | -0.02 | 33.95 | 0.40 | 9.08 | 0 | 30 | 0 | 10 |
| **30** | 0.02 | 29.15 | 0.17 | 11.43 | 0 | 30 | 0 | 10 |
| **31** | -0.07 | 13.19 | 0.20 | 32.60 | 0 | 10 | 0 | 30 |
| **32** | -0.17 | 19.64 | 0.24 | 32.07 | 0 | 10 | 0 | 30 |
| **33** | -0.19 | 19.81 | 0.28 | 31.95 | 0 | 10 | 0 | 30 |
| **34** | -0.12 | 37.65 | 0.02 | 22.06 | 0 | 50 | 0 | 10 |
| **35** | -0.13 | 31.19 | 0.01 | 25.84 | 0 | 50 | 0 | 10 |
| **36** | -0.13 | 30.00 | -0.05 | 27.48 | 0 | 50 | 0 | 10 |
| **37** | -0.20 | 16.44 | 0.30 | 47.03 | 0 | 10 | 0 | 50 |
| **38** | -0.10 | 15.11 | 0.15 | 44.70 | 0 | 10 | 0 | 50 |
| **39** | -0.15 | 16.04 | 0.22 | 46.35 | 0 | 10 | 0 | 50 |
| **40** | -0.16 | 41.76 | 0.21 | 35.81 | 0 | 50 | 0 | 30 |
| **41** | -0.12 | 45.73 | 0.24 | 32.60 | 0 | 50 | 0 | 30 |
| **42** | -0.20 | 31.02 | 0.20 | 44.84 | 0 | 50 | 0 | 30 |
| **43** | -0.05 | 22.60 | 0.51 | 49.95 | 0 | 30 | 0 | 50 |
| **44** | -0.26 | 32.65 | 0.43 | 46.47 | 0 | 30 | 0 | 50 |
| **45** | -0.12 | 29.86 | 0.47 | 47.76 | 0 | 30 | 0 | 50 |
| **46** | 9.21 | -0.39 | 0.04 | 2.67 | 10 | 0 | 0 | 0 |
| **47** | 8.72 | -0.11 | 0.03 | 3.00 | 10 | 0 | 0 | 0 |
| **48** | 8.43 | 0.67 | 0.54 | 0.06 | 10 | 0 | 0 | 0 |
| **49** | 10.98 | -0.57 | -0.31 | 10.09 | 10 | 0 | 0 | 10 |
| **50** | 9.86 | -0.30 | -0.27 | 8.86 | 10 | 0 | 0 | 10 |
| **51** | 15.21 | 0.01 | 0.17 | 6.22 | 10 | 0 | 0 | 10 |
| **52** | 30.78 | -0.13 | 0.10 | -0.03 | 30 | 0 | 0 | 0 |
| **53** | 29.25 | 0.33 | 0.09 | 1.11 | 30 | 0 | 0 | 0 |
| **54** | 31.57 | -0.17 | 0.10 | -0.50 | 30 | 0 | 0 | 0 |
| **55** | 30.98 | 0.37 | -0.06 | 32.89 | 30 | 0 | 0 | 30 |
| **56** | 33.31 | 0.01 | -0.13 | 31.88 | 30 | 0 | 0 | 30 |
| **57** | 32.09 | 0.26 | -0.19 | 31.94 | 30 | 0 | 0 | 30 |
| **58** | 46.97 | 0.41 | -0.15 | -1.39 | 50 | 0 | 0 | 0 |
| **59** | 45.90 | 0.35 | -0.17 | 2.88 | 50 | 0 | 0 | 0 |
| **60** | 47.55 | 0.23 | -0.21 | -2.70 | 50 | 0 | 0 | 0 |
| **61** | 48.94 | -1.13 | 0.03 | 48.39 | 50 | 0 | 0 | 50 |
| **62** | 49.17 | -0.97 | 0.08 | 50.08 | 50 | 0 | 0 | 50 |
| **63** | 51.72 | -0.95 | 0.09 | 46.02 | 50 | 0 | 0 | 50 |
| **64** | 30.76 | 0.55 | -0.15 | 12.83 | 30 | 0 | 0 | 10 |
| **65** | 31.25 | 0.17 | -0.08 | 10.98 | 30 | 0 | 0 | 10 |
| **66** | 31.54 | 0.22 | -0.11 | 10.71 | 30 | 0 | 0 | 10 |
| **67** | 10.74 | -1.15 | 0.74 | 38.28 | 10 | 0 | 0 | 30 |
| **68** | 8.72 | 1.51 | 1.29 | 37.63 | 10 | 0 | 0 | 30 |
| **69** | 9.54 | -0.17 | 0.71 | 32.70 | 10 | 0 | 0 | 30 |
| **70** | 46.91 | 0.94 | -0.07 | 12.95 | 50 | 0 | 0 | 10 |
| **71** | 50.73 | 1.02 | -0.08 | 9.88 | 50 | 0 | 0 | 10 |
| **72** | 49.15 | 0.93 | -0.02 | 13.98 | 50 | 0 | 0 | 10 |
| **73** | 10.60 | -1.19 | 0.80 | 40.34 | 10 | 0 | 0 | 50 |
| **74** | 10.02 | -0.56 | 0.65 | 42.86 | 10 | 0 | 0 | 50 |
| **75** | 10.40 | -0.71 | 0.77 | 47.49 | 10 | 0 | 0 | 50 |
| **76** | 49.06 | 0.92 | 0.04 | 21.67 | 50 | 0 | 0 | 30 |
| **77** | 49.78 | 0.83 | 0.03 | 23.05 | 50 | 0 | 0 | 30 |
| **78** | 49.59 | 0.22 | -0.06 | 30.68 | 50 | 0 | 0 | 30 |
| **79** | 31.69 | -0.31 | 0.02 | 49.15 | 30 | 0 | 0 | 50 |
| **80** | 31.98 | -0.37 | 0.07 | 46.90 | 30 | 0 | 0 | 50 |
| **81** | 33.27 | -0.51 | -0.08 | 49.21 | 30 | 0 | 0 | 50 |
| **82** | 0.34 | 0.68 | 9.46 | 0.16 | 0 | 0 | 10 | 0 |
| **83** | 0.34 | 0.44 | 9.40 | 0.11 | 0 | 0 | 10 | 0 |
| **84** | 0.36 | 0.38 | 9.17 | -0.17 | 0 | 0 | 10 | 0 |
| **85** | 0.03 | 0.29 | 9.82 | 9.28 | 0 | 0 | 10 | 10 |
| **86** | 0.01 | -0.06 | 9.76 | 9.25 | 0 | 0 | 10 | 10 |
| **87** | 0.00 | -0.04 | 9.87 | 9.74 | 0 | 0 | 10 | 10 |
| **88** | 0.08 | 0.29 | 30.00 | 5.35 | 0 | 0 | 30 | 0 |
| **89** | 0.28 | -0.32 | 29.82 | 2.27 | 0 | 0 | 30 | 0 |
| **90** | 0.18 | 0.75 | 29.86 | 1.72 | 0 | 0 | 30 | 0 |
| **91** | 0.02 | 0.47 | 30.34 | 30.15 | 0 | 0 | 30 | 30 |
| **92** | 0.00 | 0.49 | 30.27 | 30.53 | 0 | 0 | 30 | 30 |
| **93** | 0.01 | 0.58 | 30.17 | 30.67 | 0 | 0 | 30 | 30 |
| **94** | 0.03 | 0.03 | 49.83 | 4.51 | 0 | 0 | 50 | 0 |
| **95** | 0.10 | 0.03 | 49.71 | 3.57 | 0 | 0 | 50 | 0 |
| **96** | 0.03 | 0.12 | 50.00 | 7.71 | 0 | 0 | 50 | 0 |
| **97** | -0.13 | -0.35 | 50.37 | 50.02 | 0 | 0 | 50 | 50 |
| **98** | -0.08 | -0.48 | 49.50 | 49.28 | 0 | 0 | 50 | 50 |
| **99** | -0.10 | -0.48 | 50.15 | 49.46 | 0 | 0 | 50 | 50 |
| **100** | -0.03 | 0.17 | 31.20 | 8.83 | 0 | 0 | 30 | 10 |
| **101** | 0.01 | 0.03 | 30.81 | 7.73 | 0 | 0 | 30 | 10 |
| **102** | 0.02 | 0.11 | 30.82 | 8.30 | 0 | 0 | 30 | 10 |
| **103** | 0.20 | 0.16 | 10.02 | 30.01 | 0 | 0 | 10 | 30 |
| **104** | 0.20 | 0.18 | 10.06 | 29.96 | 0 | 0 | 10 | 30 |
| **105** | 0.20 | 0.16 | 10.35 | 30.05 | 0 | 0 | 10 | 30 |
| **106** | -0.06 | -0.07 | 49.83 | 3.80 | 0 | 0 | 50 | 10 |
| **107** | -0.09 | 0.00 | 49.84 | 6.39 | 0 | 0 | 50 | 10 |
| **108** | -0.09 | -0.01 | 49.94 | 6.73 | 0 | 0 | 50 | 10 |
| **109** | -0.14 | -0.29 | 10.12 | 49.96 | 0 | 0 | 10 | 50 |
| **110** | -0.23 | 0.04 | 10.34 | 49.91 | 0 | 0 | 10 | 50 |
| **111** | -0.36 | 0.36 | 10.82 | 49.93 | 0 | 0 | 10 | 50 |
| **112** | 0.00 | 0.08 | 49.93 | 16.17 | 0 | 0 | 50 | 30 |
| **113** | -0.04 | -0.06 | 50.35 | 23.36 | 0 | 0 | 50 | 30 |
| **114** | -0.01 | -0.03 | 50.12 | 21.50 | 0 | 0 | 50 | 30 |
| **115** | -0.39 | -0.73 | 30.45 | 50.76 | 0 | 0 | 30 | 50 |
| **116** | -0.41 | -0.49 | 30.45 | 50.82 | 0 | 0 | 30 | 50 |
| **117** | -0.44 | -0.48 | 31.06 | 51.71 | 0 | 0 | 30 | 50 |
| **118** | 0.09 | 13.00 | 10.07 | 10.68 | 0 | 10 | 10 | 10 |
| **119** | 0.21 | 9.78 | 10.01 | 10.01 | 0 | 10 | 10 | 10 |
| **120** | 0.10 | 9.26 | 10.06 | 10.63 | 0 | 10 | 10 | 10 |
| **121** | 0.13 | 21.11 | 9.99 | 18.58 | 0 | 10 | 10 | 30 |
| **122** | 0.35 | 12.03 | 9.33 | 31.47 | 0 | 10 | 10 | 30 |
| **123** | 0.05 | 17.43 | 9.55 | 23.93 | 0 | 10 | 10 | 30 |
| **124** | 0.51 | 30.54 | 9.88 | 8.77 | 0 | 30 | 10 | 10 |
| **125** | 0.34 | 26.78 | 10.32 | 11.90 | 0 | 30 | 10 | 10 |
| **126** | 0.28 | 26.42 | 10.36 | 8.69 | 0 | 30 | 10 | 10 |
| **127** | 0.14 | 9.82 | 29.81 | 10.32 | 0 | 10 | 30 | 10 |
| **128** | 0.12 | 9.89 | 30.02 | 10.22 | 0 | 10 | 30 | 10 |
| **129** | 0.13 | 9.88 | 30.03 | 10.26 | 0 | 10 | 30 | 10 |
| **130** | 0.58 | 28.51 | 8.39 | 30.31 | 0 | 30 | 10 | 30 |
| **131** | 0.70 | 30.55 | 7.64 | 33.11 | 0 | 30 | 10 | 30 |
| **132** | 0.58 | 30.44 | 8.31 | 29.99 | 0 | 30 | 10 | 30 |
| **133** | 0.01 | 22.64 | 30.43 | 17.65 | 0 | 30 | 30 | 10 |
| **134** | 0.01 | 22.56 | 30.46 | 17.71 | 0 | 30 | 30 | 10 |
| **135** | 0.00 | 20.81 | 30.20 | 19.57 | 0 | 30 | 30 | 10 |
| **136** | -0.01 | 19.33 | 30.08 | 20.76 | 0 | 10 | 30 | 30 |
| **137** | -0.02 | 16.96 | 29.70 | 22.96 | 0 | 10 | 30 | 30 |
| **138** | -0.01 | 18.79 | 29.88 | 21.26 | 0 | 10 | 30 | 30 |
| **139** | -0.21 | 29.92 | 29.74 | 29.86 | 0 | 30 | 30 | 30 |
| **140** | -0.22 | 30.35 | 29.62 | 29.51 | 0 | 30 | 30 | 30 |
| **141** | -0.19 | 30.31 | 29.58 | 29.43 | 0 | 30 | 30 | 30 |

**
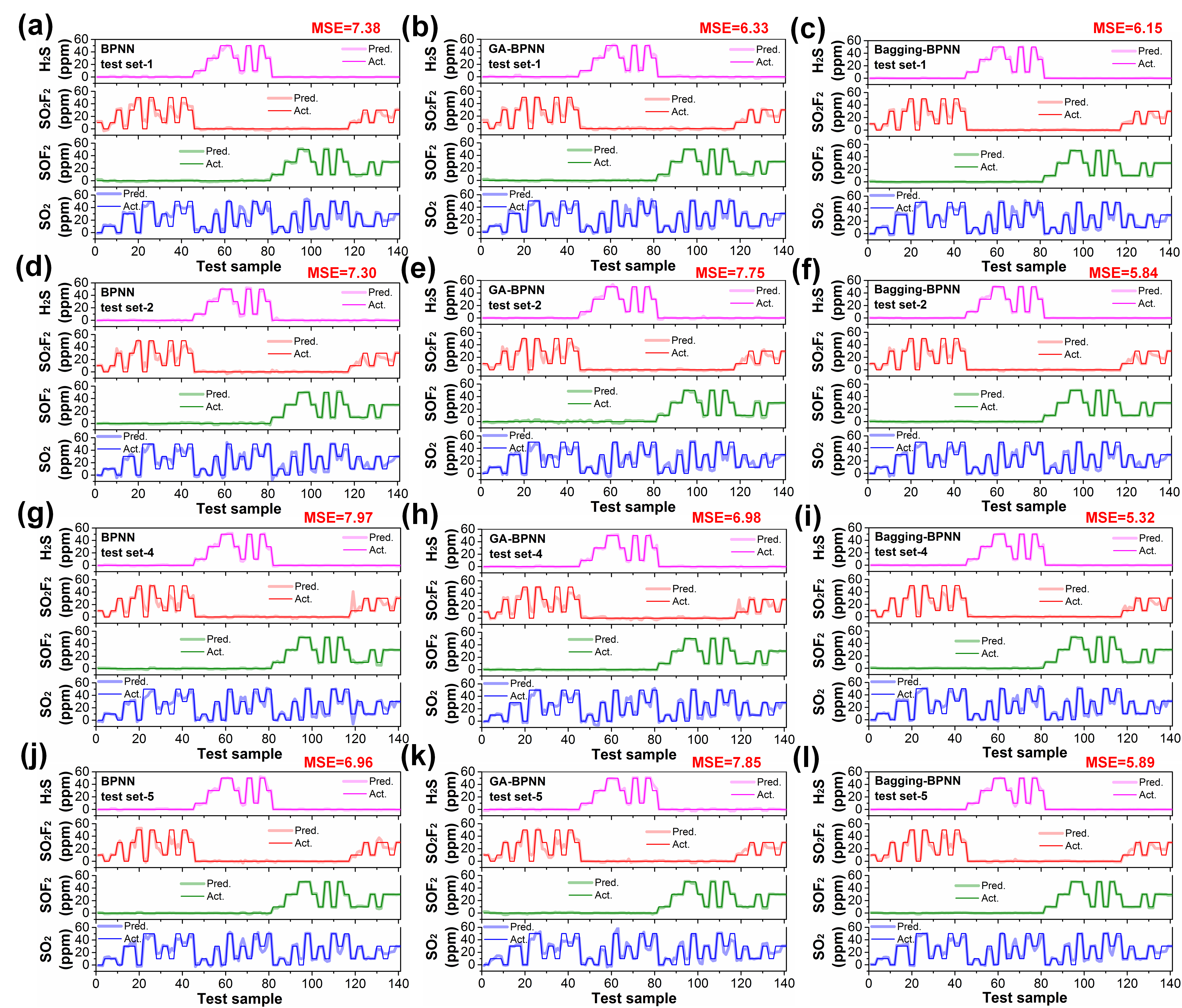
**

**Fig. S19** For the first cross-validation dataset, the regressions to predict the concentrations of gas mixtures via (a) BPNN, (b) GA-BPNN and (c) Bagging-BPNN. For the second cross-validation dataset, the regressions to predict the concentrations of gas mixtures via (d) BPNN, (e) GA-BPNN and (f) Bagging-BPNN. For the fourth cross-validation dataset, the regressions to predict the concentrations of gas mixtures via (g) BPNN, (h) GA-BPNN and (i) Bagging-BPNN. For the fifth cross-validation dataset, the regressions to predict the concentrations of gas mixtures via (j) BPNN, (k) GA-BPNN and (l) Bagging-BPNN.

**
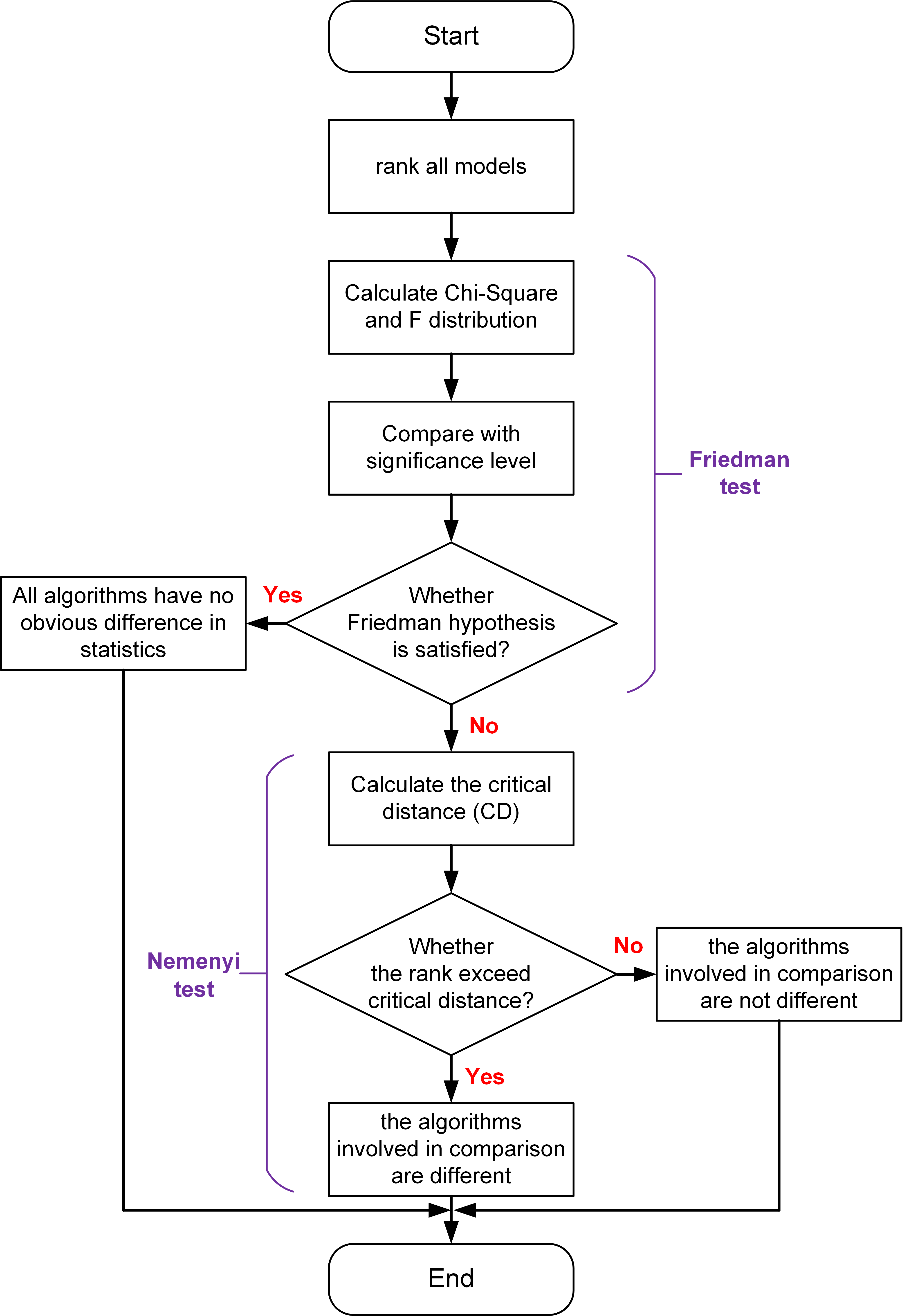
**

**Fig. S20** The flow chart of Friedman test and Nemenyi post-hoc test.

**Table S3** The actual concentrations of the unknown dataset.

| **Group** | **Number of unknown dataset** | **H_2_S (ppm)** | **SO_2_F_2_ (ppm)** | **SOF_2_ (ppm)** | **SO_2_ (ppm)** |
| --- | --- | --- | --- | --- | --- |
| **Test 1** | **1** | 0 | 10 | 0 | 0 |
|  | **2** | 0 | 10 | 0 | 0 |
|  | **3** | 0 | 10 | 0 | 10 |
|  | **4** | 0 | 10 | 0 | 10 |
|  | **5** | 0 | 30 | 0 | 0 |
|  | **6** | 0 | 30 | 0 | 0 |
|  | **7** | 0 | 30 | 0 | 30 |
|  | **8** | 0 | 30 | 0 | 30 |
|  | **9** | 0 | 50 | 0 | 0 |
|  | **10** | 0 | 50 | 0 | 0 |
|  | **11** | 0 | 30 | 0 | 50 |
|  | **12** | 0 | 30 | 0 | 50 |
| **Test 2** | **13** | 10 | 0 | 0 | 0 |
|  | **14** | 10 | 0 | 0 | 0 |
|  | **15** | 30 | 0 | 0 | 0 |
|  | **16** | 30 | 0 | 0 | 0 |
|  | **17** | 30 | 0 | 0 | 30 |
|  | **18** | 30 | 0 | 0 | 30 |
|  | **19** | 30 | 0 | 0 | 50 |
|  | **20** | 30 | 0 | 0 | 50 |
| **Test 3** | **21** | 0 | 0 | 10 | 0 |
|  | **22** | 0 | 0 | 10 | 0 |
|  | **23** | 0 | 0 | 30 | 0 |
|  | **24** | 0 | 0 | 30 | 0 |
|  | **25** | 0 | 0 | 30 | 30 |
|  | **26** | 0 | 0 | 30 | 30 |
|  | **27** | 0 | 0 | 30 | 50 |
|  | **28** | 0 | 0 | 30 | 50 |
| **Test 4** | **29** | 0 | 30 | 30 | 30 |
|  | **30** | 0 | 30 | 30 | 30 |

**Table S4** The rank of different algorithms for the unknown dataset with various noise.

| **Dataset (noisy)** | **SDAE-KNN** | **SDAE-Ada** | **SDAE-SVM** | **SDAE-BPNN** | **SDAE-GA** | **SDAE-Bag** | **PCA-**  **KNN** | **PCA-**  **Ada** | **PCA-**  **SVM** | **PCA-**  **BPNN** | **PCA-**  **GA** | **PCA-**  **Bag** |
| --- | --- | --- | --- | --- | --- | --- | --- | --- | --- | --- | --- | --- |
| **None** | 7 | 4 | 1.5 | 5 | 6 | 3 | 8 | 11 | 1.5 | 12 | 10 | 9 |
| **1%** | 7 | 3 | 1 | 4 | 6 | 2 | 8 | 11 | 5 | 12 | 10 | 9 |
| **3%** | 6 | 5 | 1 | 4 | 7 | 2 | 8 | 12 | 3 | 10 | 11 | 9 |
| **5%** | 5 | 8 | 1 | 4 | 6 | 3 | 7 | 12 | 2 | 10 | 11 | 9 |
| **7%** | 4 | 3 | 1 | 6 | 7 | 2 | 8 | 12 | 5 | 11 | 10 | 9 |
| **10%** | 5 | 7 | 1 | 4 | 6 | 2 | 8 | 11 | 3 | 12 | 9 | 10 |
| **Aver.** | 5.67 | 5.00 | 1.08 | 4.50 | 6.33 | 2.33 | 7.83 | 11.50 | 3.25 | 11.17 | 10.17 | 9.16 |

**S3. Mixed gas experiment under different humidity**

Under various humid background (25%, 33%, 50%, 75%), the mixing scheme of SO_2_F_2_ and SO_2_ is shown in **Table S5**. In fact, introducing the impact of humidity is equivalent to adding another measured gas, and this problem can be transformed into the identification of three gas components. The methods of feature extraction and dimensionality reduction still follow previous strategies. Firstly, construct the feature matrix from the original signals of GS microchip. In **Fig. S19**, we can observe the sample clustering in PCA space. It can be found that all kinds of samples have obvious discrimination at low relative humidity (25%). However, with the increase of humidity (33% and 50%), the discrimination of various kinds of samples in the PCA space gradually decreases.

Similarly, 15 samples for each atmosphere were cyclically recorded, so there is a total of 405 samples for 27 detected gases. Randomly select 20% of the samples as test set, and the remaining samples are used for training and validation. The parameter settings of SDAE-based models are the same as those in **Table 2**. The training curve and the error regression curve of SDAE are shown in **Fig. S20**. For the humid test set, the prediction results by different recognition algorithms are shown in **Fig. S21**, where the method of Bagging-BPNN performs the highest classification accuracy.

**Table S5** Under various humid background (25%, 33%, 50%, 75%), the mixing scheme of SO_2_F_2_ and SO_2_.

| **Experiment** | **Label** | **RH (%)** | **SO_2_F_2_ (ppm)** | **SO_2_ (ppm)** |
| --- | --- | --- | --- | --- |
| **SO_2_F_2_, SO_2_** | 1 | 25.5 | 0 | 10 |
|  | 2 | 25.5 | 10 | 0 |
|  | 3 | 25.5 | 10 | 10 |
|  | 4 | 25.5 | 0 | 30 |
|  | 5 | 25.5 | 30 | 0 |
|  | 6 | 25.5 | 30 | 30 |
|  | 7 | 25.5 | 30 | 10 |
|  | 8 | 25.5 | 10 | 30 |
|  | 9 | 33.7 | 0 | 10 |
|  | 10 | 33.7 | 10 | 0 |
|  | 11 | 33.7 | 10 | 10 |
|  | 12 | 33.7 | 0 | 30 |
|  | 13 | 33.7 | 30 | 0 |
|  | 14 | 33.7 | 30 | 30 |
|  | 15 | 33.7 | 30 | 10 |
|  | 16 | 33.7 | 10 | 30 |
|  | 17 | 50 | 0 | 10 |
|  | 18 | 50 | 10 | 0 |
|  | 19 | 50 | 10 | 10 |
|  | 20 | 50 | 0 | 30 |
|  | 21 | 50 | 30 | 0 |
|  | 22 | 50 | 30 | 30 |
|  | 23 | 50 | 30 | 10 |
|  | 24 | 50 | 10 | 30 |
|  | 25 | 76.5 | 0 | 10 |
|  | 26 | 76.5 | 10 | 0 |
|  | 27 | 76.5 | 10 | 10 |


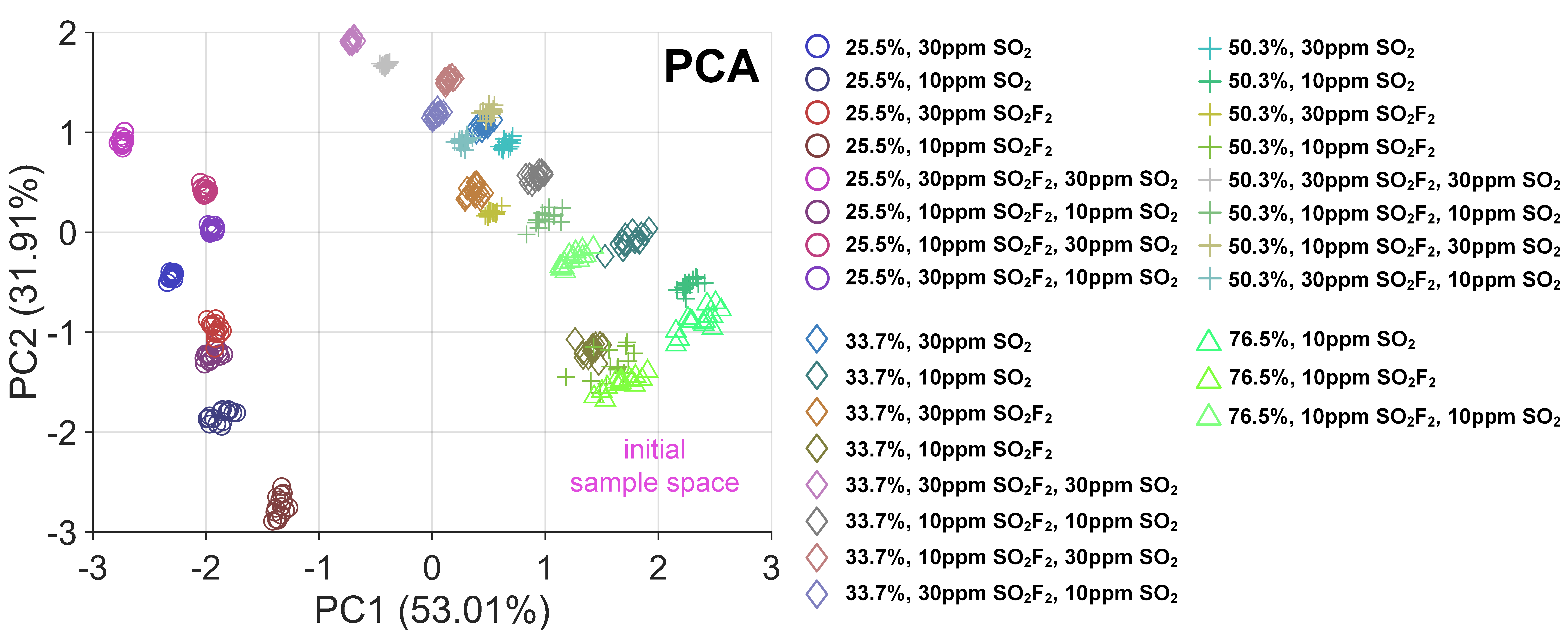


**Fig. S21** The visualization of humidity samples in PCA space.


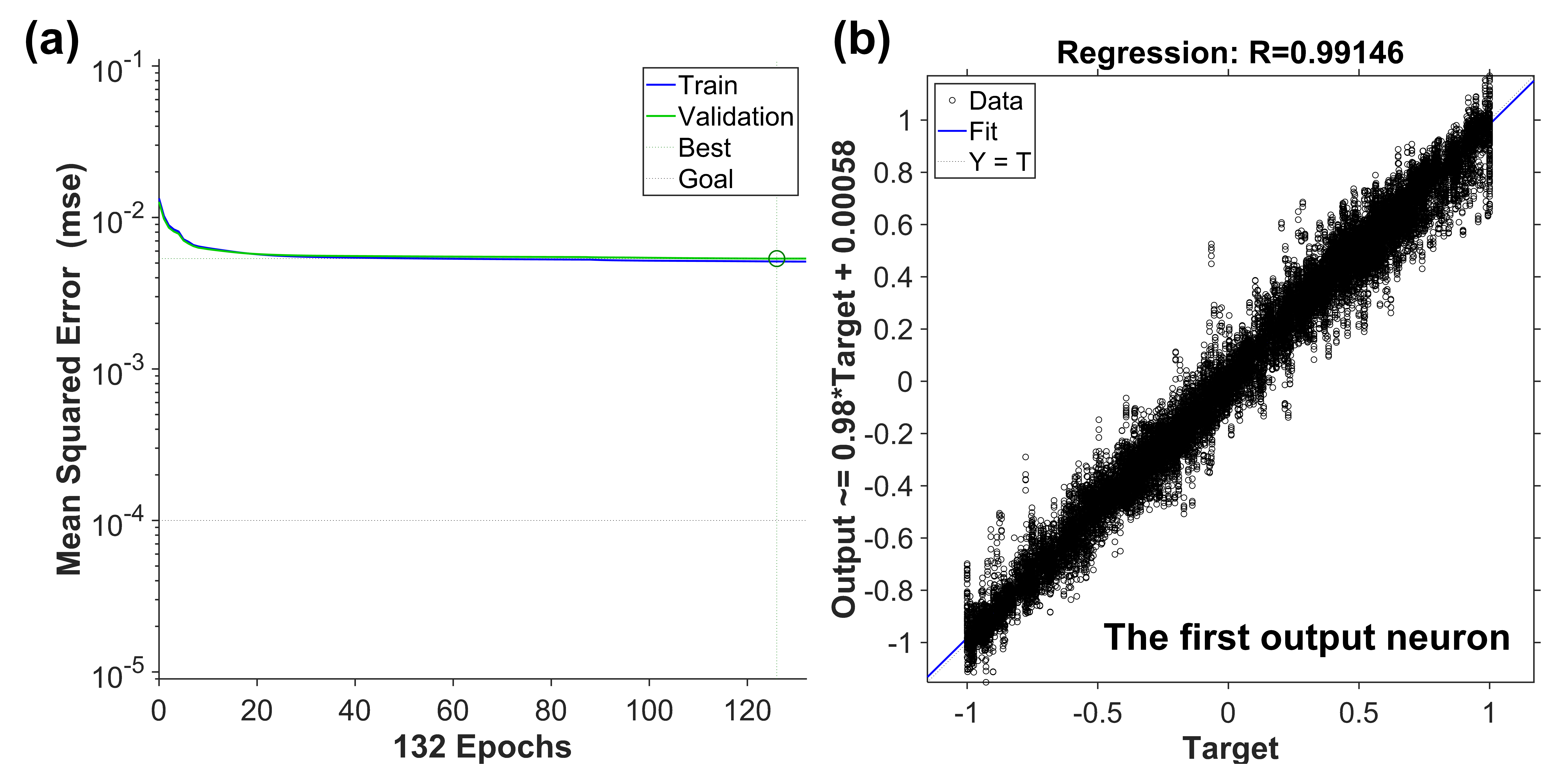


**Fig. S22** (a) The SDAE training curve. (b) The error regression curves of the first neuron in output layer.





**Fig. S23** For the humidity test set, the confusion graphics of (a) KNN, (b) AdaBoost decision tree, (c) SVM, (d) BPNN, (e) GA-BPNN, (f) Bagging-BPNN.

**S4. Mixed gas experiment with great difference in concentration**

The additional experiments are supplemented to investigate whether the MGS sensor could quantify gas mixtures with large differences in concentrations or not. Four less toxic gases (CH_4_, H_2_, C_2_H_2_ and CO) with great difference in concentrations, were chosen as the measured gases. Their calibration concentrations were 500 ppm, 10 ppm, 250 ppm and 25 ppm, respectively. Apart from gas sources, the test system and recognition algorithms were the same as those in the manuscript. Gas mixing schemes of four gases are presented in **Table S6**, where their concentrations differ by orders of magnitude. In this process, 15 cyclic tests were collected at each atmosphere. Therefore, the dataset containing 150 samples can be achieved for 10 measured gases.

**Table S6** Gas mixing schemes of CH_4_, H_2_, C_2_H_2_ and CO.

| **Label** | **CH_4_ (ppm)** | **H_2_ (ppm)** | **C_2_H_2_ (ppm)** | **CO (ppm)** |
| --- | --- | --- | --- | --- |
| **1** | 500 | 0 | 0 | 0 |
| **2** | 0 | 10 | 0 | 0 |
| **3** | 0 | 0 | 250 | 0 |
| **4** | 0 | 0 | 0 | 25 |
| **5** | 500 | 10 | 0 | 0 |
| **6** | 0 | 10 | 250 | 0 |
| **7** | 500 | 0 | 0 | 25 |
| **8** | 500 | 10 | 250 | 0 |
| **9** | 500 | 10 | 0 | 25 |
| **10** | 500 | 10 | 250 | 25 |

In **Fig. S24**, five cycling response-recovery curves are presented to verify the repeatability of GS microchip upon exposure to 10 gas mixtures. Obviously, there exists transient differences upon different measured gases, which provide the basis for further gas recognition. **Fig. S24a-S24d** present the results of the device for testing single gas. It can be concluded that, the GS microchip is more sensitive to CO than CH_4_, and ZIF8-WO_3_ gets the lowest response among these three materials. As the gas composition changes, the responses of GS microchip changes accordingly. When four gases are mixed, the response values of the three sensors all increase significantly (**Fig. S24j**).


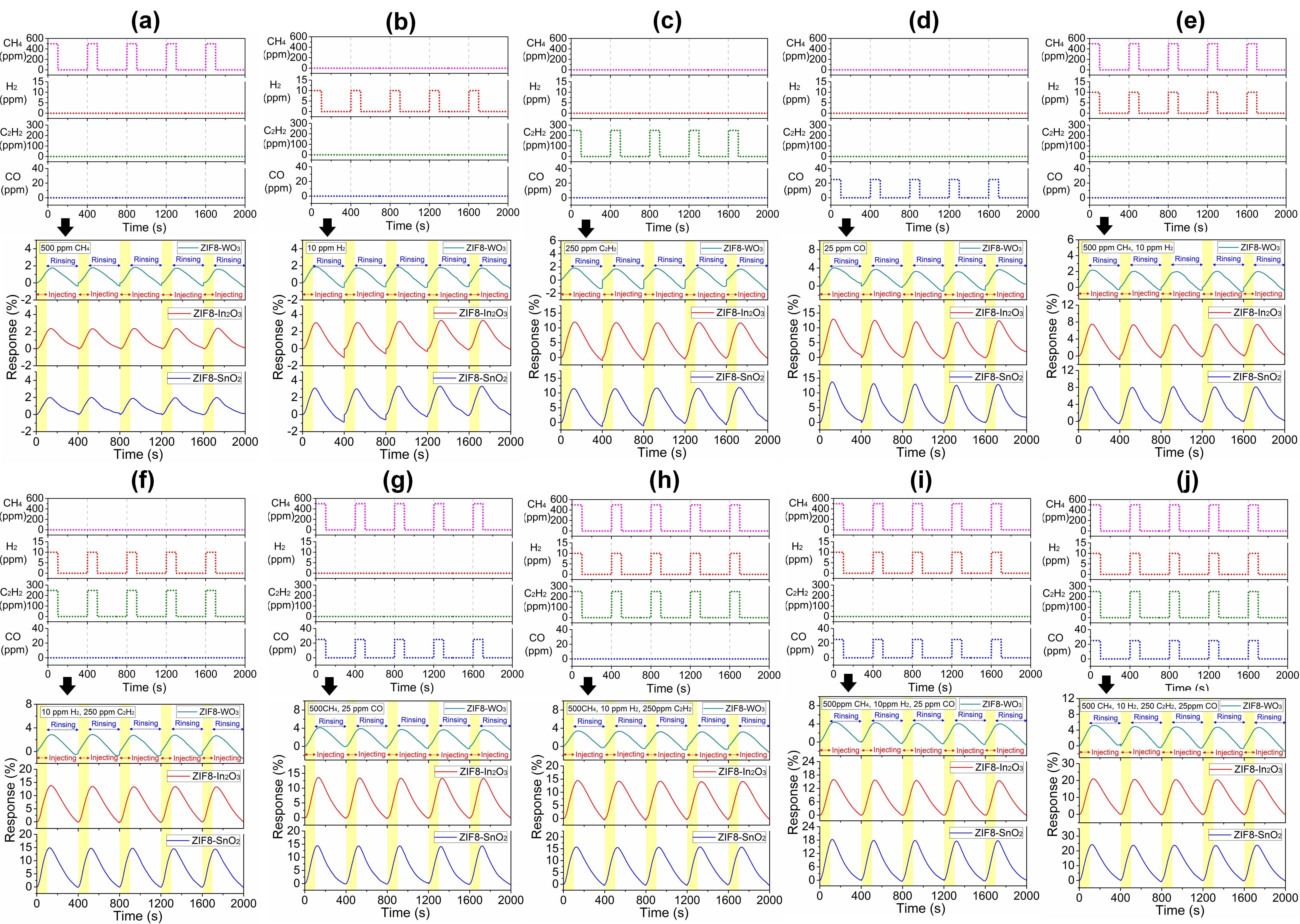


**Fig. S24** The original signals of the GS microchip. Respectively exposed to **a** 500 ppm CH_4_, **b** 10 ppm H_2_, **c** 250 ppm C_2_H_2_, **d** 25 ppm CO, **e** 500 ppm CH_4_ and 10 ppm H_2_, **f** 10 ppm H_2_ and 250 ppm C_2_H_2_, **g** 500 ppm CH_4_ and 25 ppm CO, **h** 500 ppm CH_4_, 10 ppm H_2_ and 250 ppm C_2_H_2_, **i** 500 ppm CH_4_, 10 ppm H_2_ and 25 ppm CO, **j** 500 ppm CH_4_, 10 ppm H_2_, 250 ppm C_2_H_2_ and 25 ppm CO.

The methods of feature extraction and dimensionality reduction still follow previous strategies. In **Fig. S25**, we can observe the sample clustering in PCA space. It can be found that most samples for various gases have more obvious discrimination. But for the region of 25 ppm CO, it’s overlapped with that of 500 ppm CH_4_ and 25 ppm CO, which means that the response of GS microchip is more affected by CO.





**Fig. S25** The visualization of samples with additional gases in PCA space.

For each gas mixing scheme, 15 samples were recorded cyclically. Thus, there has a total of 150 samples for 10 detected gases. 20% of the samples are randomly selected as the test set, and the remaining samples are employed for training and validation. The parameter settings of SDAE-based models are the same as those in **Table 2**. The training curve and the error regression curve of SDAE are shown in **Fig. S26**.


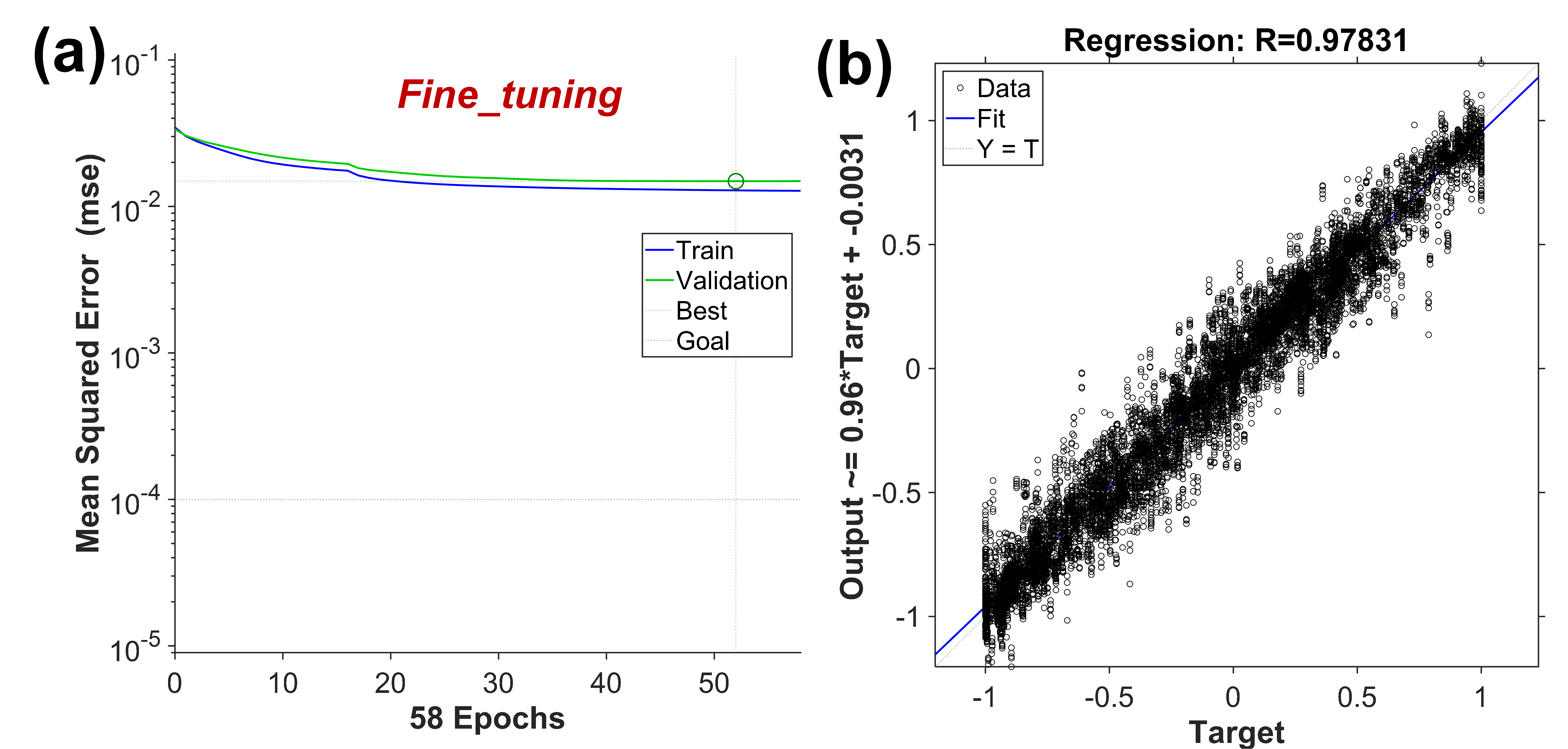


**Fig. S26** (a) The SDAE training curve. (b) The error regression curves of the first neuron in output layer.

For the selected test set, the prediction results by different recognition algorithms are shown in **Fig. S27**. Among the models, Bagging-BPNN achieves the highest classification accuracy (96.67%), which proves the performance enhancement of single BPNN (90%). For three classification algorithms, their accuracy hasn’t exceeded 90%. Besides, the regression results of three models based on BPNN are shown in **Fig. S27g-S27i**, where the higher overlap between the predicted and actual values means the algorithm is more accurate. From the point of view of mean square error (MSE), Bagging-BPNN (73.53) has indeed achieved higher performance than BPNN (134.15) and GA-BPNN (76.45).

Through supplementing the experiments for quantifying four gas mixtures (CH_4_, H_2_, C_2_H_2_ and CO), we find that the method proposed in this manuscript has strong universality. Even if mixing four gases, the GS microchip still has ability to recognize. Moreover, the experiments with orders of magnitude difference in gas concentration (500ppm, 10ppm, 250ppm, 25ppm) also proved that, our GS microchip could quantify gas mixtures with very different concentrations.


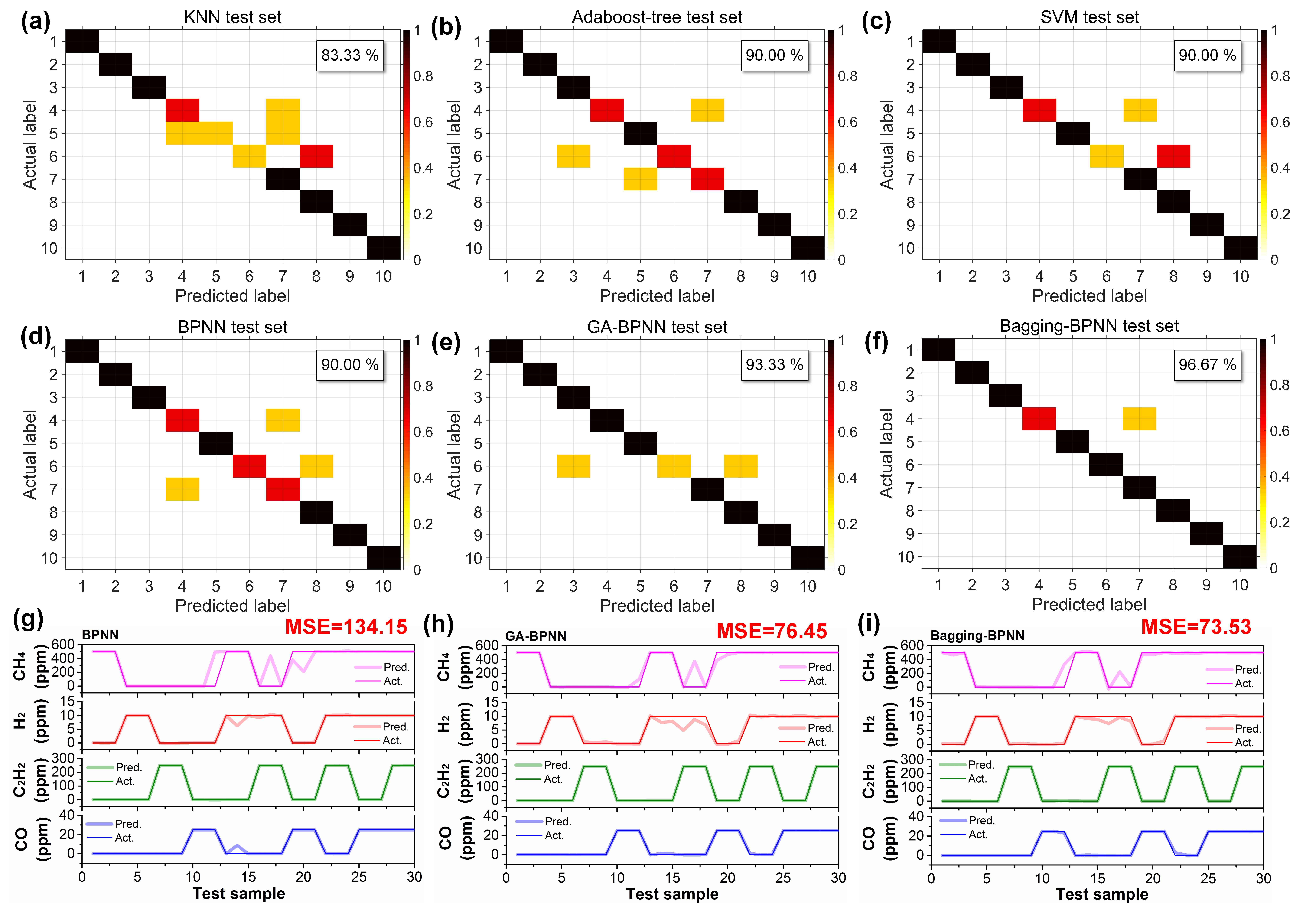


**Fig. S27** Gas recognition with machine learning algorithms. For the selected test set, the confusion matrix of **a** KNN, **b** AdaBoost decision tree, **c** SVM, **d** BPNN, **e** GA-BPNN, **f** Bagging-BPNN. The regressions to predict the concentrations of gas mixtures via **h** BPNN, **i** GA-BPNN and **j** Bagging-BPNN.
